# SynAI: an AI-driven cancer drugs synergism prediction platform

**DOI:** 10.1093/bioadv/vbad160

**Published:** 2023-11-10

**Authors:** Kuan Yan, Runjun Jia, Sheng Guo

**Affiliations:** Data Science and Bioinformatics, Crown Bioscience, Suzhou, Jiangsu Province 215000, P.R. China; Data Science and Bioinformatics, Crown Bioscience, Suzhou, Jiangsu Province 215000, P.R. China; Data Science and Bioinformatics, Crown Bioscience, Suzhou, Jiangsu Province 215000, P.R. China

## Abstract

**Summary:**

The SynAI solution is a flexible AI-driven drug synergism prediction solution aiming to discover potential therapeutic value of compounds in early stage. Rather than providing a finite choice of drug combination or cell lines, SynAI is capable of predicting potential drug synergism/antagonism using *in silico* compound SMILE (Simplified Molecular Input Line Entry System) sequences. The AI core of SynAI platform has been trained against cell lines and compound pairs listed by NCI (National Cancer Institute)-Almanac and DurgCombDB datasets. In total, the training data consists of over 1 200 000 *in vitro* synergism tests on 150 cancer cell lines of different organ origins. Each cell line is tested against over 6000 pairs of FDA (Food and Drug Administration) approved compound combinations. Given one or both candidate compound in SMILE sequence, SynAI is able to predict the potential Bliss score of the combined compound test with the designated cell line without the needs of compound synthetization or structural analysis; thus can significantly reduce the candidate screening costs during the compound development. SynAI platform demonstrates a comparable performance to existing methods but offers more flexibilities for data input.

**Availability and implementation:**

The evaluation version of SynAI is freely accessible online at https://synai.crownbio.com.

## 1 Introduction

**Figure 1.**
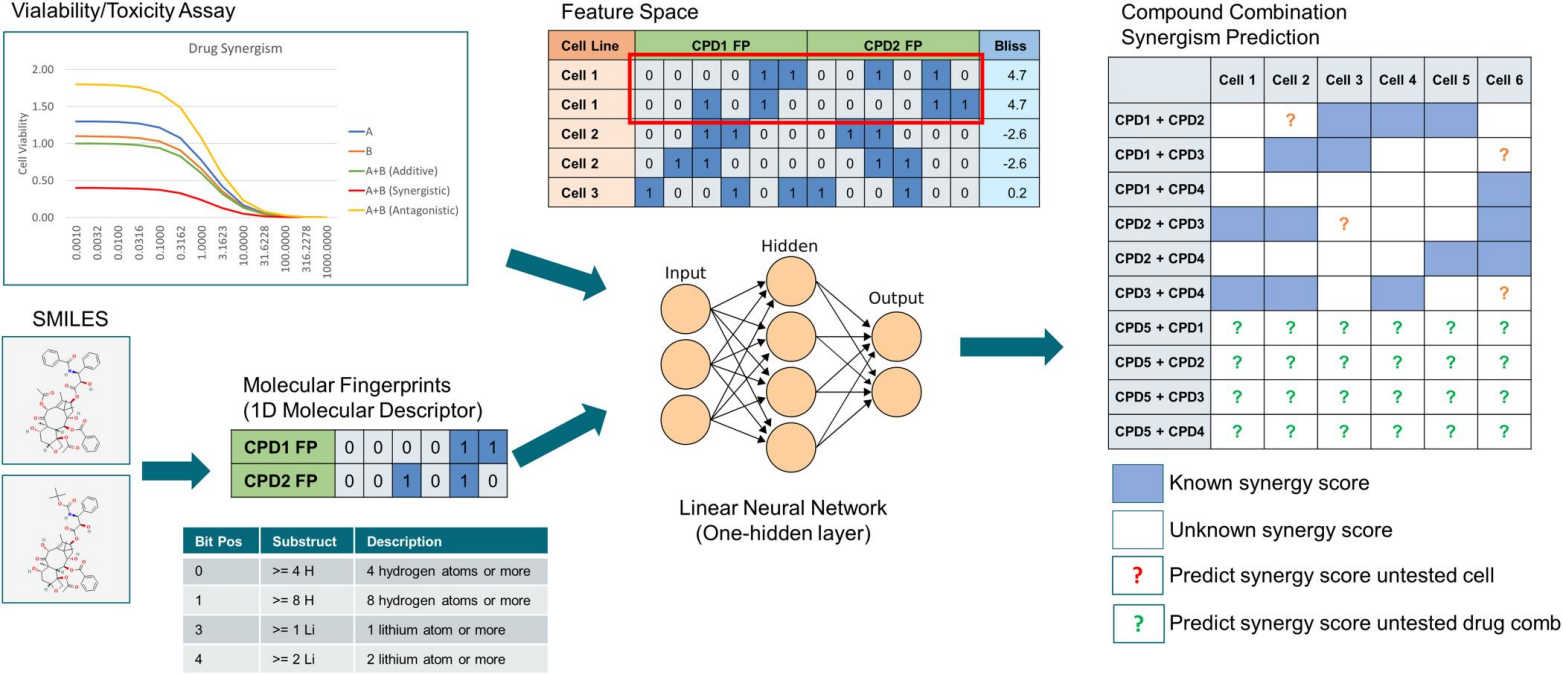
Principal design of SynAI platform.

It is a common practice to administrate multiple drugs simultaneously during a cancer treatment session to create a combined effect greater than individual drug potencies, known as drug synergistic effect. Searching for synergistic drug combinations can increase the therapeutic efficiency of existing standard-of-care (SOC) and investigational drugs (Jaaks *et al.* 2022). It is frequently accomplished via large-scale *ex vivo* high-throughput viability screening. However, the *ex vivo* approach is often laborious and resource intensive. For a synergism screening of merely 100 compounds with single PDX model, it would have required an assay setup of minimum 60 × 384-well plates and it would take months to complete the assay. Contrary to experimental approach, computational algorithms for predicting drug synergy can alleviate the limitation by providing an initial sift of potentially synergistic drug combinations for the later experimental validations (Liu and Zhao 2016, Holbeck *et al.* 2017, Preuer *et al.* 2018, Sidorov *et al.* 2019, Kuenzi *et al.* 2020, An *et al.* 2022). However, existing synergism prediction platforms are largely limited to a predefined set of compounds or cell lines (Preuer *et al.* 2018, Kuenzi *et al.* 2020). In this work, we introduce the SynAI platform which utilizes the drug SMILE (Simplified Molecular Input Line Entry System) sequence as direct inputs crossing multiple cell models for a more flexible synergism prediction setup (cf. Fig. 1).

SynAI design offers several advantages compared to existing synergistic analysis solutions:

1. Utilizing only SMILE sequences as inputs, no compound synthetization is required for the analysis. Thus, SynAI can significantly decrease the costs of candidate screening.
2. In addition, SynAI requires no compound structural info thus further reduces the early screening duration. The prediction is genuine *in silico.*
3. Finally, the AI core of SynAI is designed to be retrainable which can be updated with new experiment data in future.

**Figure 2.**
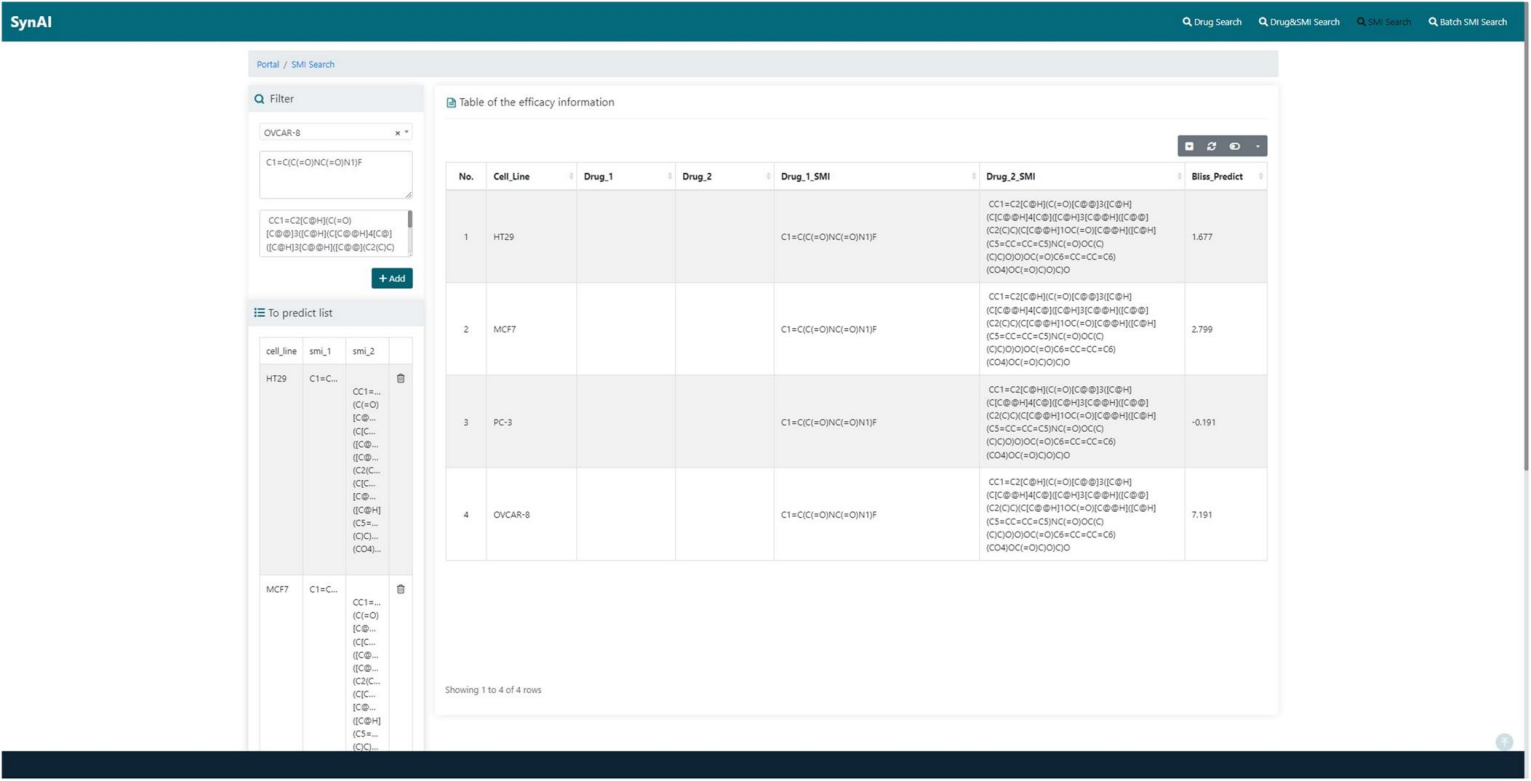
open Access web-UI of SynAI.

The latest version of SynAI provides four input modes to meet different research interests (cf. Fig 2) (cf. Table 1). An API (Application Programming Interface) is being added allowing FASTA-like data file to be upload for bulk processing.

**Table 1.** SynAI prediction mode list.

SynAI prediction modes	Input 1	Input 2	Cell
Drug versus Drug	Single drug	Single drug	Multicell
Drug versus SMILE	Single drug	Single SMILE	Multicell
SMI versus SMI	Single SMILE	Single SMILE	Multicell
SMI bulk run	SMILE seq file		Multicell

## 2 Materials and methods

The AI core of SynAI platform was constructed (cf. Fig. 1) using the MLP (multi-layer perceptron) network under the PyTorch framework (Paszke *et al.* 2019). Instead of having one neural network trained against all cell lines and drug combinations, one MLP network was trained for each cell line (cf. Fig. 5). Essentially, these networks predict Bliss score (Liu and Zhao 2016, Yang *et al.* 2020) for any combination of SMILE-based feature sets known as the molecular fingerprints (Pedregosa *et al.* 2011, Wu *et al.* 2018). The neural networks were trained against the National Cancer Institute (NCI)-Almanac database (cf. Fig. 7) (Holbeck *et al.* 2017) and the learnability of SynAI was verified using both NCI-Almanac and DrugCombDB (cf. Fig. 8) (Liu *et al.* 2020). During the training, a hyperparameter tuning (HT) study (cf. Fig. 6) was performed for SynAI AI core and other benchmark algorithms (RNN, RF, GBX); allowing an objective comparison of algorithm performances.

### 2.1 Data preparation

SynAI platform starts with two popular *ex vivo* synergism datasets: (i) NCI-Almanac database (Holbeck *et al.* 2017) is frequently referred as the synergism benchmark dataset constructed with a systematic evaluation of *ex vivo* therapeutic activity of over 5000 pairs of Food and Drug Administration (FDA)-approved cancer drugs against a panel of 60 well-characterized human tumor cell lines (NCI-60) to uncover combinations with greater than additive growth-inhibitory activity. In total, over 300K of compound pairs are covered in NCI dataset and (ii) DrugCombDB (Liu *et al.* 2020) is a newly developed collective drug synergism database by integrating multiple drug synergism datasets. Similar to NCI-data, DrugCombDB houses over 450K drug pairs over 120 cell lines. On average, each cell line was tested against over 8000 pairs of compounds.

**Figure 3.**
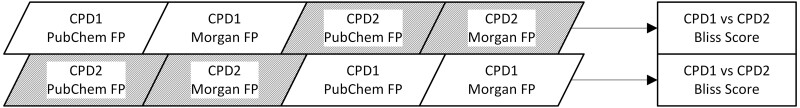
Elaboration of feature space and target space, a duplicated entry with inverted input order is created for the training to make sure the same bliss score is produced regardless of compound input order.


To prepare the data for SynAI network training, each small-molecule drug in the dataset was first converted into molecular fingerprints based on its SMILE sequence. Two fingerprint frameworks, namely PubChem[881-bit] (Kim *et al.* 2022) and Morgan-1D[1024-bit] (Capecchi *et al.* 2020) were utilized by SynAI to provide more coverage of chemical properties of the compound. These drug fingerprints were generated using the Scikit library (Pedregosa *et al.* 2011). The collection of fingerprint data was used as the input data to model against the Bliss score (Liu and Zhao 2016, Yang *et al.* 2020). During hyperparameter tuning study, the possible combination of the molecular fingerprint is also considered as one hyperparameter and the conclusion suggested the PubChem+Morgan1D combination provides the most resilient performance crossing various setups. The molecular fingerprints of both input compounds are concatenated to create the feature space for the network training (cf. Fig. 3), thus the dimensionality of the final feature space becomes:

$$2\times \left(\mathrm{le}n_{\mathrm{PubChemFP}}+\mathrm{le}n_{\mathrm{Morgan}1D\mathrm{FP}}\right),$$

where len_PubChemFP = 881 and len_Morgan1DFP = 1024. Finally, the target space consists of the experimental bliss score provided by the datasets. Furthermore, the input order (CPD1+CPD2) of compound fingerprints is also inverted to create a secondary entry to simulate an inverted input pattern (CPD2 + CPD1). With both patterns used as the training inputs, the trained network is forced to produce the same bliss score regardless the input order of compounds (cf. Fig. 3).

**Figure 4.**
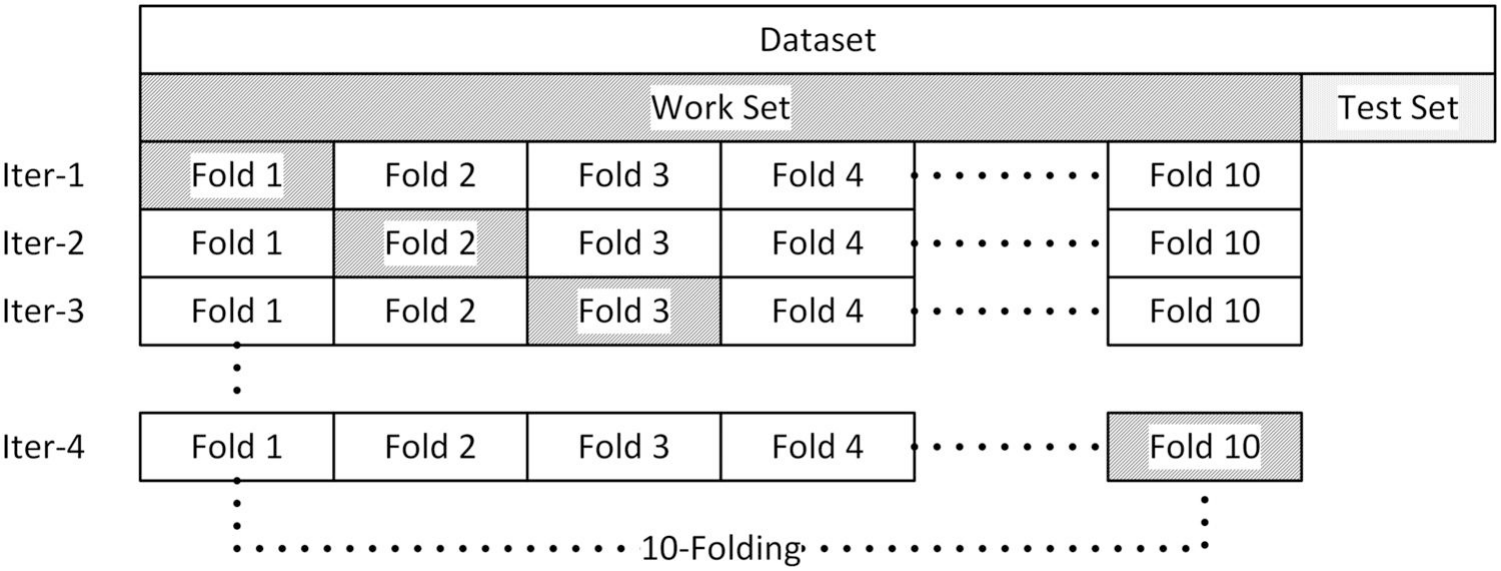
Illustration of data splits for a 10-fold cross validation.

For training validation, the converted dataset is first split into 90% working set and 10% testing set. The working set is further k-folded into k-combinations of 90% training set and 10% validating set. Such a cross-validation strategy is often referred as the 80/10/10 splits (cf. Fig. 4), but in implementation the dataset is split into 81/9/10.

During each iteration of the k-folding, one new network is trained using the training set. The trained network is further evaluated with the validating set. The network with the highest validating set performance is selected as the final (best) network. The final network will be evaluated against the testing set which is recognized as the final performance of the trained network.

### 2.2 Model training

**Figure 5.**
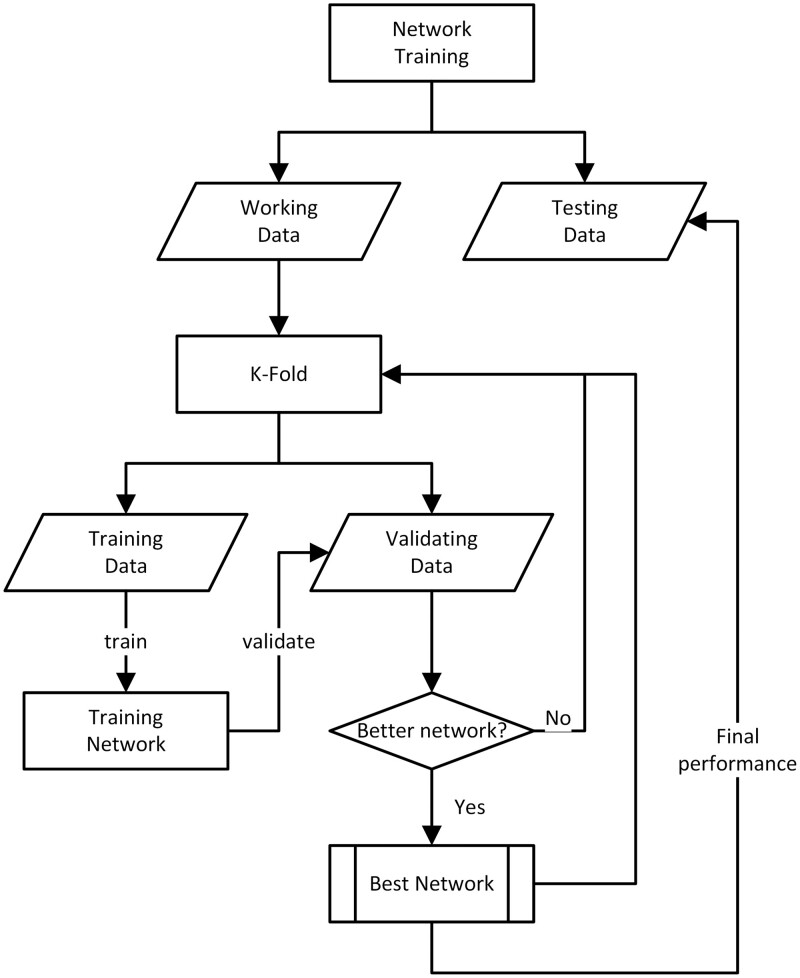
Network training procedure employed by SynAI platform.

For the network design, we started with an arbitrary one-layer MLP network (Russel and Norvig 2012) for its ability to avoid both overfitting and underfitting (cf. sup 2.2.1). The network training workflow is elaborated in Fig. 5. The NCI-Almanac dataset is split using k-folding cross validation following the popular 80/10/10 strategy. From the iterations, the best performed network based on validation set PCC (Pearson cross correliation) is selected as the final network. The 10% independent test set will be used to estimate the final performance of the network.

**Figure 6.**
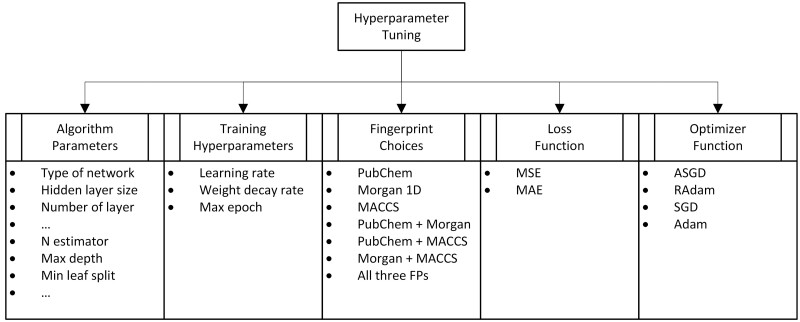
Hyperparameter tuning setup for SynAI core training. For machine learning algorithms such as random forest, similar hyperparameter tuning tests were also performed. The network parameters are replaced with random forest parameters such as maximum depth, number of estimators, number of features and minimum leaf split.

When compared to other deep learning frameworks, MLP is structurally simpler and resilient to overfitting phenomena (Russel and Norvig 2012, Wu *et al.* 2018). It is often the first choice for table-like data classification or regression study whereas CNN (Convolutional Neural Networks) often with image inputs and RNN (Recurrent Neural Networks) with sequential inputs. From the initial MLP network training, it is notable that the choice of hyperparameters (cf. Fig. 6) and training setups can significantly influence the performance of network. Moreover, the combinations of different molecular fingerprints also play an essential role in algorithm performance. Thus, a set of hyperparameter tuning tests were performed (cf. Fig. 6) with NCI dataset (Holbeck *et al.* 2017). To provide an objective comparison with the benchmark algorithms, the hyperparameter tuning test was also performed for RNN, random forest (RF), and gradient boosting search (GBX) (Russel and Norvig 2012), respectively.

The output of these tuning tests showed that the combination of single-layer MLP with RAdam (Rectified Adam) and MSE (Mean Squared Error) provided the most stable and accurate prediction performance (cf. Table 2 and Supplementary Data 2.2.1). The RNN showed a strong overfit tendency but overall provides similar performance as MLP (cf. Supplementary Data 2.2.2). The RF and GBX algorithms (cf. Supplementary Data 2.2.3) both provide decent performance as well but slightly lower than MLP and RNN solution. The test further showed that the increasing number of hidden layers did improve the training performance of the MLP network (cf. Supplementary Data 2.2). However, it does not consequentially improve the corresponding testing performance, thus showing a strong tendency of overfitting. In summary, the hyperparameter tuning test results suggest that with the optimal parameter setting, the deep learning solutions (MLP and RNN) show slightly stronger performance compared to traditional machine learning solutions (RF and GBX).

**Table 2.** Final testing set PCC (Pearson cross correlation) of algorithms with NCI dataset.

PCC score	Cell lines		
Algorithm	MCF7	OVCAR-8	SK-MEL-5
SynAI	0.68 ± 0.02	0.56 ± 0.07	0.86 ± 0.02
RF	0.64 ± 0.02	0.55 ± 0.03	0.89 ± 0.02
GBX	0.66 ± 0.02	0.48 ± 0.05	0.88 ± 0.02
RNN	0.54 ± 0.12	0.43 ± 0.06	0.83 ± 0.08

**Figure 7.**
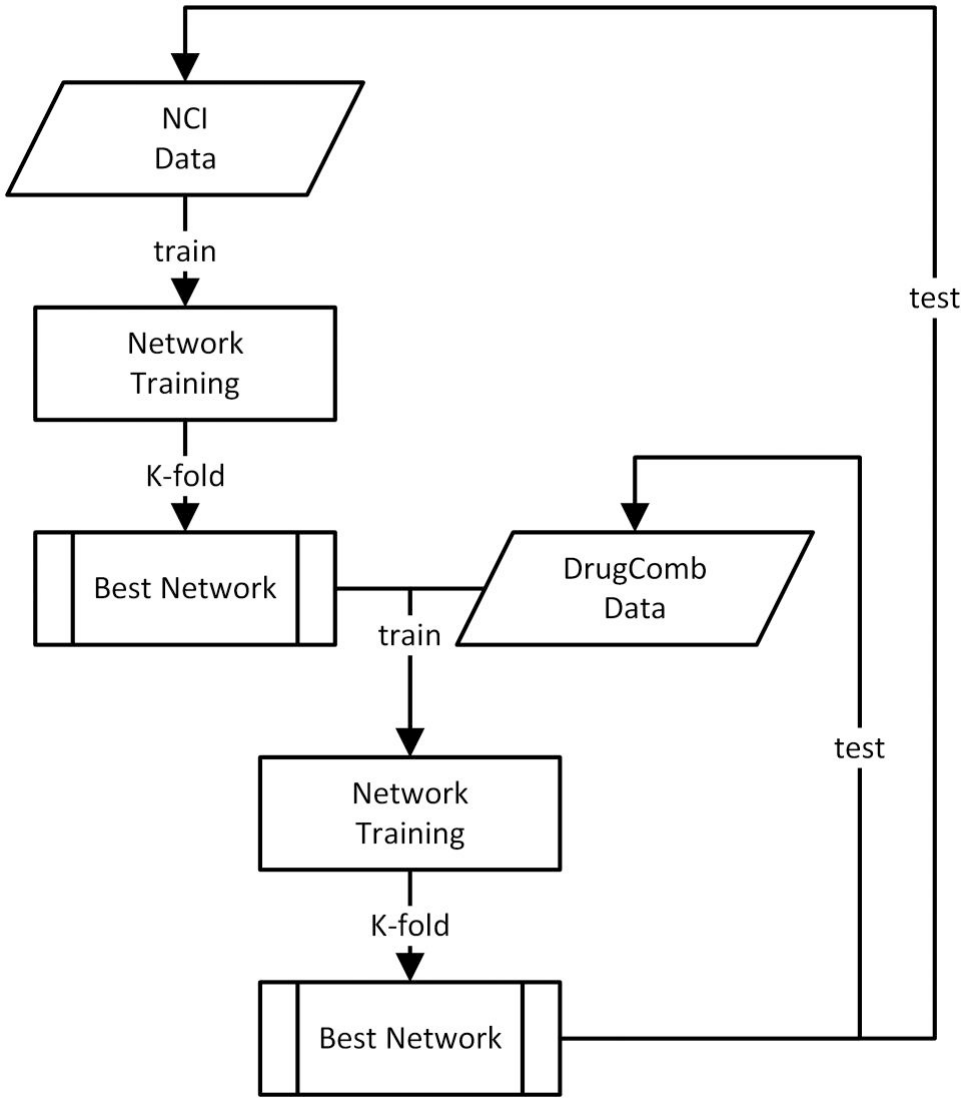
Workflow of NCI-first-DrugCombDB-second strategy.

**Figure 8.**
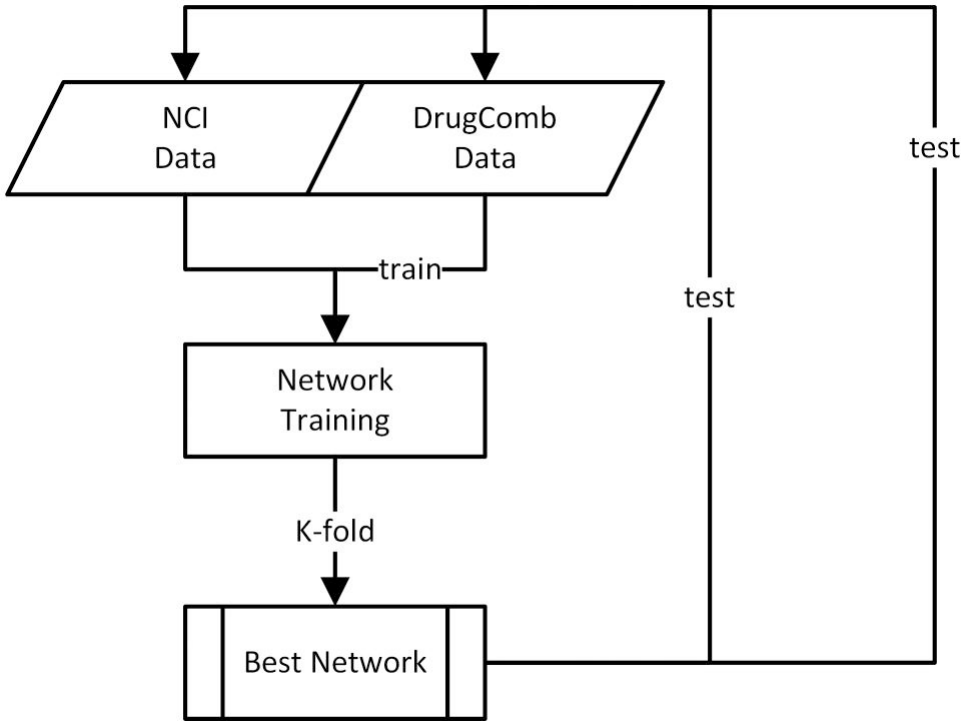
Workflow of NCI+DrugCombDB combined strategy, two data sources are first combined as a single dataset. Instead of normal *n*-fold which is completely random, a stratified *n*-fold based on dataset name is employed to make sure samples were drawn proportionally to both datasets.

To further test the learnability of SynAI, the best network from NCI training rounds was retrained with DrugCombDB using the similar strategy. The difference in retraining round is that instead of using a newly initialized network, the best network from NCI training round will be used as the initial network for DrugCombDB training round (cf. Fig. 7). The goal is to simulate the capability of updating existing networks with new data. When comparing to combining all data together (cf. Fig. 8) for training, the early experiment shows that retraining of network in fact yields higher performance in general (cf. Supplementary Data 2.3.1).

Here we would like to emphasize that in the next section the reported results of all algorithms are based on the best performance hyperparameter set and training setups from the hyperparameter tuning tests.

### 2.3 Model validation

To compare different algorithms (cf. Supplementary Data 2.1), we chose Pearson correlation coefficient (PCC) (Preuer *et al.* 2018, Sidorov *et al.* 2019) between measured and predicted Bliss scores as the universal performance criteria (Sidorov *et al.* 2019, An *et al.* 2022). Here the utilization of PCC score should not to be confused with the objective function (also known as loss function or metrics) for the algorithm training procedure. The reason is due to that different algorithms may utilize different objective function (MSE, R-squared, PCC or other derived score systems) during training that makes it difficult to compare the final model performances crossing different algorithms or training strategies. Choosing a mutual yet independent score can provide an objective conclusion over the algorithm performances.

**Figure 9.**
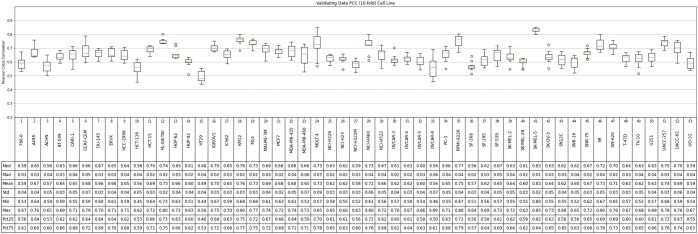
The final validating set Pearson cross coefficient (PCC) between real bliss score and predicted bliss score per-cell line at epoch = 2048 from all cross-validation iterations (NCI-data).

**Figure 10.**
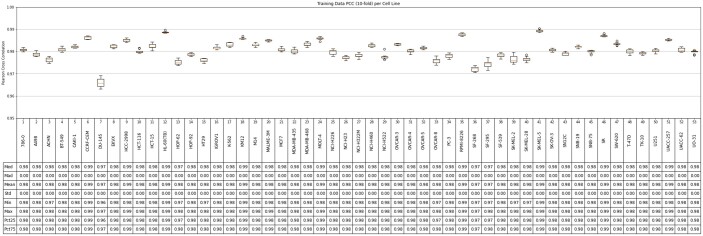
The final training set Pearson cross coefficient (PCC) between real bliss score and predicted bliss score per-cell line of from all cross-validation iterations (NCI-data).

**Figure 11.**
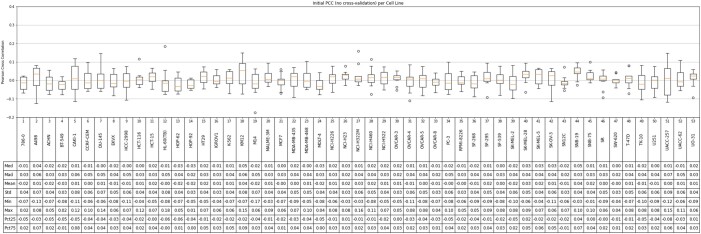
The epoch = 0 Pearson cross coefficient (PCC) between real bliss score and predicted bliss score per-cell line from all cross-validation iterations (NCI-data). The result confirms no reusing of model occurred during training procedure at each iteration of *n*-folding.

Based on the cross-validation experiments, SynAI yielded a test PCC between 0.55 and 0.88 for different cell lines with the NCI-Almanac dataset (cf. Fig. 9). The initial PCC (cf. Fig. 11) confirmed that no reusing or retraining of the network were performed during each iteration of the k-folding cross-validation. In addition, the training PCC of SynAI reached 0.99 ± 0.01 (cf. Fig. 10). Such performance is consistent with the reported performance provided by other research groups (Liu and Zhao 2016, Preuer *et al.* 2018, Sidorov *et al.* 2019, Kuenzi *et al.* 2020, An *et al.* 2022). Moreover, the NCI dataset (Holbeck *et al.* 2017) reported an interclass correlation coefficient ICC = 0.71, which is translated as the maximum correlation observed between replicas of the same experiment conditions. Theoretically, the regression model cannot achieve a final performance in PCC higher than the experiment ICC score (Holbeck *et al.* 2017, Sidorov *et al.* 2019). Thus, ICC is often recognized as the maximum performance a regression model can achieve with the dataset.

### 2.4 Additional studies

During our early evaluation of training setups, there were several experimental outcomes which we believe provided insights of the training setups.

**Figure 12.**
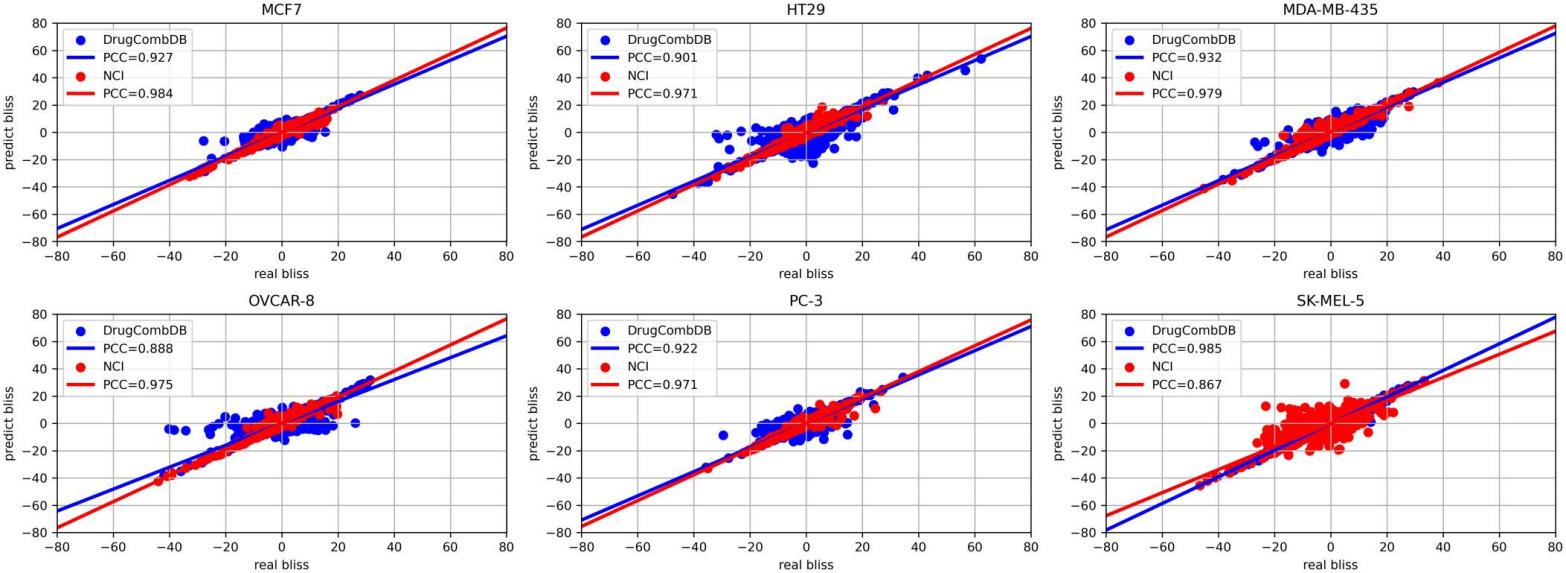
Per-cell line validation set performance when NCI and DrugCombDB were combined as the data source.

**Multi-dataset training** (cf. Fig. 12 and Supplementary Data 2.3.1**)**: In this experiment, we train per-cell networks using combined data from NCI and DrugCombDB. The NCI + DrugCombDB is treated as a single dataset and split into per-cell data for network training. The goal of this test is to check if our proposed solution (retraining strategy) can yield similar performance if comparing to data-combining strategy. The results show that the combined dataset yields a lower performance compared to the retraining strategy.

**Figure 13.**
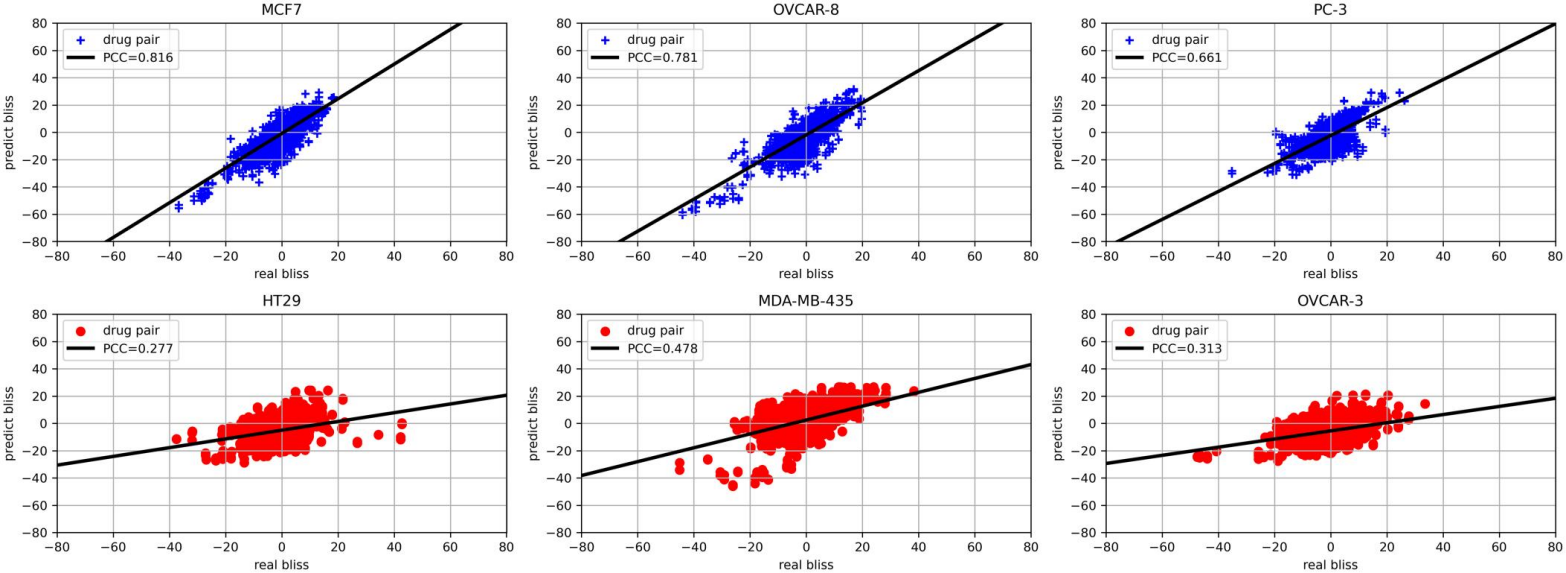
Per-cell line validation set performance if single-model is trained to cover all cell lines.

**Single-network training** (cf. Fig. 13 and Supplementary Data 2.3.2**)**: In this experiment, we trained a single network using the whole NCI without splitting into per-cell data. The goal is to check if per-cell training is indeed necessary as suggested by existing literatures (Preuer *et al.* 2018, Kuenzi *et al.* 2020). Due to the memory consumption, we included six random cell lines from NCI-data to run such tests. The output shows that the trained network prediction performance is dominated by few cell lines while the other cell lines’ performance is relatively low.

## 3 Conclusion

Compared to existing literatures, SynAI yields a similar performance in all categories (training, validating, and testing) but provides more flexibilities for data input by using directly the SMILE sequence of drug. In addition, the computational core of SynAI can be constantly updated with new experiment inputs from different cell lines and drug combinations. Its adaptive and dynamic nature allows the SynAI platform to learn from new data feeds from future studies.

## Supplementary Material

vbad160_Supplementary_Data
