# Supplementary material for: SynAI: an AI-driven cancer drugs synergism prediction platform
Source: Bioinform Adv. 2023 Nov 10;3(1):vbad160. doi: 10.1093/bioadv/vbad160 (PMC10660295; doi:10.1093/bioadv/vbad160)
Supplement: vbad160_Supplementary_Data [file vbad160_supplementary_data.pdf]

SynAI Supplementary Data

S1. SynAI Development Environment

S1.1 Language and IDE

The SynAI is developed using **PyCharm (Community Edition 2022)** with **Python 3.7** environment. The core dependency library of SynAI is PyFingerprint which is a customized library producing various molecular descriptors (molecular fingerprint).

The running of the SynAI is limited to Python 3.7 due to the dependent package **openbabel 3.0.0** can only work under Python 3.7. The openbabel 3.0.0 is a dependent library for the PyFingerprint package.

The following packages are directly employed by SynAI. For secondary layer packages, please referring to the individual package details.

via PyPI

- numpy==1.21.6 (https://pypi.org/project/numpy/1.21.6/)
- pandas==1.1.5 (https://pypi.org/project/pandas/1.1.5/)
- openbabel==3.0.0 (https://pypi.org/project/openbabel/3.0.0/)
    - OpenBabel software (https://openbabel.org/wiki/Category:Installation)
    - Visual C++ 2010 Redistribution (https://www.microsoft.com/en-us/download/details.aspx?id=26999)
- scikit-learn==0.21.3 (https://pypi.org/project/scikit-learn/0.21.3/
- tqdm==4.64.0 (https://pypi.org/project/tqdm/4.64.0/)
- Pytorch (https://pytorch.org/get-started/locally/)
    - torch==1.13.0+cu116
    - torchvision==0.14.0+cu116
    - torchaudio==0.13.0cu+116
- RDKit
    - rdkit==2022.9.2 (https://pypi.org/project/rdkit/2022.9.2/)
    - rdkit-pypi==2022.3.5 (https://pypi.org/project/rdkit-pypi/2022.3.5/)
- pubchempy==1.0.1 (https://pypi.org/project/PubChemPy/1.0.1/)
- smile==0.1.0 (https://pypi.org/project/smile/0.1.0/)

Non-standard package

- PyFingerprint (https://github.com/hcji/PyFingerprint)

S2. SynAI Model Performance Evaluation

S2.1 Preliminary Design and Evaluation of SynAI Core Model

The defintion of DL model is elaborated as below. Due to the genetic variation discussed in early literatures (An2022), DL model is trained for each cell line. During the training of the DL model, a n-fold cross-validation is performed to understand the essential performance of SynAI model (cf. Fig.1). In the early experiment we observed a strong tendency of model being overfit (cf. Fig.2), thus an additional hyperparameter tuning test was performed to understand the influence of parameters on the final model performance (cf. Fig.2 ~ 4).

```python
# design for SynAI L1 network
class L1(nn.Module):
    """
    singe-layer perceptron network
    """
    def __init__(self, input_size, amp=2, dropout=0.5, bias=False):
        super(L1, self).__init__()

        n_size = int(amp * input_size)  # hidden layer amplification

        self.layer = nn.Sequential(
            nn.Linear(input_size, n_size, bias=bias),  # first layer
            nn.Dropout(p=dropout),  # drop out layer avoid overtraining
            nn.ReLu(),  # converge layer
            nn.Linear(n_size, 1, bias=bias)  # output layer
        )

    def forward(self, x):
        return self.layer(x)
```

```python
# design for SynAI L1 network
class L2(nn.Module):
    """
    2-layer perceptron network
    """
    def __init__(self, input_size, amp=2, dropout=0.5, bias=False):
        super(L2, self).__init__()

        n_size = int(amp * input_size)  # hidden layer amplification

        self.layer = nn.Sequential(
            nn.Linear(input_size, n_size, bias=bias),  # layer-1
            nn.Dropout(p=dropout),  # drop out layer avoid overtraining
            nn.Linear(n_size, n_size, bias=bias),  # layer-2
            nn.ReLu(),  # converge layer
            nn.Linear(n_size, 1, bias=bias)  # output layer
        )

    def forward(self, x):
        return self.layer(x)
```

```python
# design for SynAI L1 network
class L4(nn.Module):
    """
    4-layer perceptron network
    """
    def __init__(self, input_size, amp=2, dropout=0.5, bias=False):
        super(L4, self).__init__()

        n_size = int(amp * input_size)  # hidden layer amplification

        self.layer = nn.Sequential(
            nn.Linear(input_size, n_size, bias=bias),  # layer-1
            nn.Dropout(p=dropout),  # drop out layer avoid overtraining
            nn.Linear(n_size, n_size, bias=bias),  # layer-2
            nn.Linear(n_size, n_size, bias=bias),  # layer-3
            nn.Linear(n_size, n_size, bias=bias),  # layer-4
            nn.ReLu(),  # converge layer
            nn.Linear(n_size, 1, bias=bias)  # output layer
        )

    def forward(self, x):
        return self.layer(x)
```

Fig.1

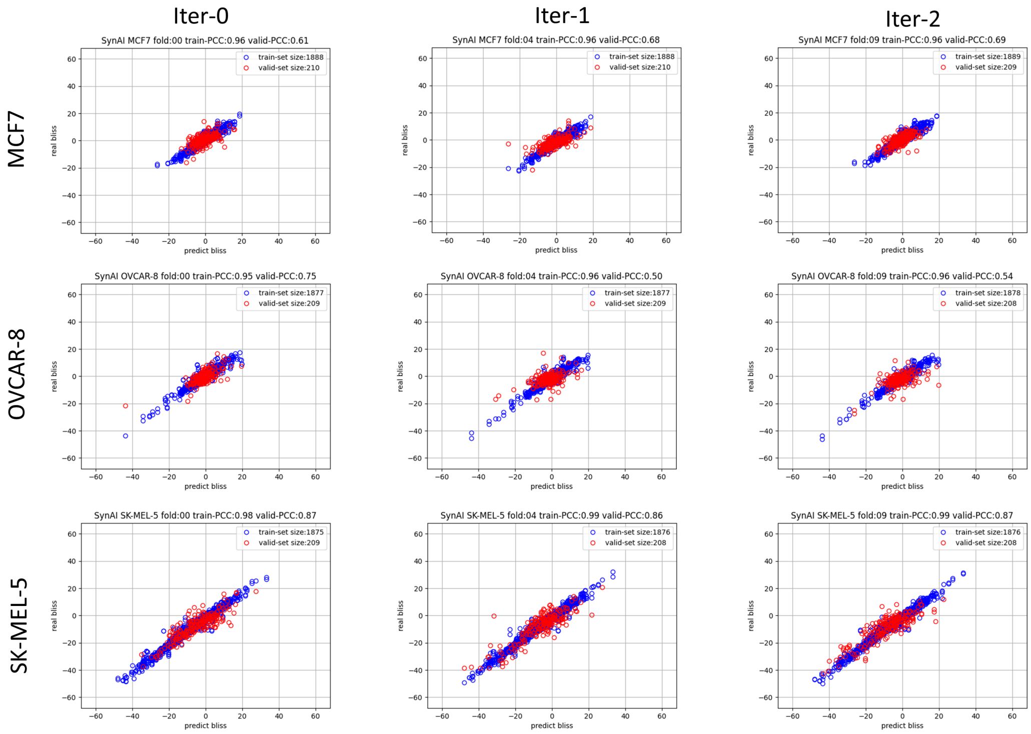

Fig.1 fitting visualization of training and validation set in each iteration of cross-validation at different cell line. Each mark represents a combination of two PDA-approved chemo-compound for cancer treatment. In general each cell line responses differently to the treatment. For example, treatment potentcy is overal stronger in SK-MEL-5 cell line while much weaker in OVCAR-8.

Initital PCC (Epoch=0)

The PCC of epoch=0 (cf. Fig.1) shows that all initial bliss score has no correlation to real bliss score. The data also shows that our model trianing procedure was properly initalized in each cross-validation iteration. There is no reusing of trained model from other iteration.

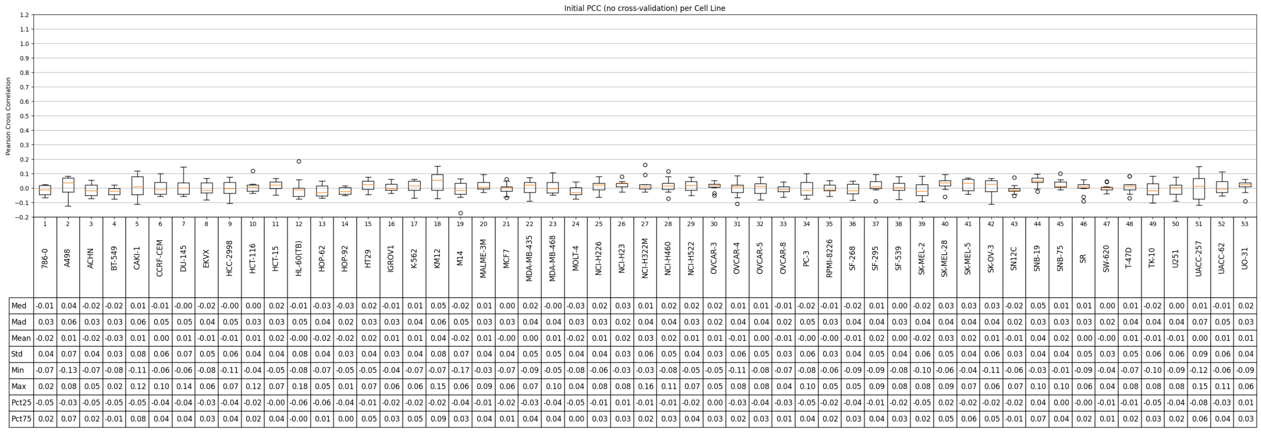

	1 786-0	2 A498	3 ACHN	4 BT-549	5 CAKI-1	6 CCRF-CEM	7 DU-145	8 EKVX	9 HCC-2998	10 HCT-116	11 HCT-15	12 HL-60(TB)	13 HOP-62	14 HOP-92	15 HT29	16 IGROV1	17 K-562	18 KM12
Med	-0.01	0.04	-0.02	-0.02	0.01	-0.01	-0.00	-0.02	-0.00	0.00	0.02	-0.01	-0.03	-0.03	0.02	-0.01	0.01	0.05
Mad	0.03	0.06	0.03	0.03	0.06	0.05	0.05	0.04	0.05	0.03	0.03	0.05	0.04	0.02	0.03	0.03	0.04	0.06
Mean	-0.02	0.01	-0.02	-0.03	0.01	0.00	0.01	-0.01	-0.01	0.01	0.02	-0.00	-0.02	-0.02	0.02	0.01	0.01	0.04
Std	0.04	0.07	0.04	0.03	0.08	0.06	0.07	0.05	0.06	0.04	0.04	0.08	0.04	0.03	0.04	0.03	0.04	0.08
Min	-0.07	-0.13	-0.07	-0.08	-0.11	-0.06	-0.06	-0.08	-0.11	-0.04	-0.05	-0.08	-0.07	-0.05	-0.05	-0.04	-0.07	-0.07
Max	0.02	0.08	0.05	0.02	0.12	0.10	0.14	0.06	0.07	0.12	0.07	0.18	0.05	0.01	0.07	0.06	0.06	0.15
Pct25	-0.05	-0.03	-0.05	-0.05	-0.05	-0.04	-0.04	-0.03	-0.04	-0.02	-0.00	-0.06	-0.06	-0.04	-0.01	-0.02	-0.02	-0.02
Pct75	0.02	0.07	0.02	-0.01	0.08	0.04	0.04	0.03	0.04	0.02	0.04	-0.00	0.01	0.00	0.05	0.03	0.05	0.09

	19 M14	20 MALME-3M	21 MCF7	22 MDA-MB-435	23 MDA-MB-468	24 MOLT-4	25 NCI-H226	26 NCI-H23	27 NCI-H322M	28 NCI-H460	29 NCI-H522	30 OVCAR-3	31 OVCAR-4	32 OVCAR-5	33 OVCAR-8	34 PC-3	35 RPMI-8226	36 SF-268
Med	-0.02	0.01	0.00	0.02	-0.00	-0.03	0.02	0.03	0.01	0.02	0.02	0.02	0.01	0.01	-0.01	-0.02	-0.01	-0.02
Mad	0.05	0.03	0.03	0.04	0.04	0.03	0.03	0.02	0.04	0.04	0.03	0.02	0.04	0.04	0.02	0.05	0.03	0.04
Mean	-0.02	0.01	-0.00	0.00	0.01	-0.02	0.01	0.02	0.03	0.02	0.01	0.01	-0.01	0.00	-0.01	-0.00	-0.00	-0.01
Std	0.07	0.04	0.04	0.05	0.05	0.04	0.04	0.03	0.06	0.05	0.04	0.03	0.06	0.05	0.03	0.06	0.03	0.04
Min	-0.17	-0.03	-0.07	-0.09	-0.05	-0.08	-0.06	-0.03	-0.03	-0.08	-0.05	-0.05	-0.11	-0.08	-0.07	-0.08	-0.06	-0.09
Max	0.06	0.09	0.06	0.07	0.10	0.04	0.08	0.08	0.16	0.11	0.07	0.05	0.08	0.08	0.04	0.10	0.05	0.05
Pct25	-0.04	-0.01	-0.02	-0.03	-0.04	-0.05	-0.01	0.01	-0.01	-0.01	-0.02	0.00	-0.03	-0.04	-0.03	-0.05	-0.02	-0.04
Pct75	0.03	0.04	0.01	0.04	0.04	0.00	0.03	0.03	0.02	0.03	0.04	0.03	0.02	0.03	0.01	0.04	0.02	0.03

	37 SF-295	38 SF-539	39 SK-MEL-2	40 SK-MEL-28	41 SK-MEL-5	42 SK-OV-3	43 SN12C	44 SNB-19	45 SNB-75	46 SR	47 SW-620	48 T-47D	49 TK-10	50 U251	51 UACC-257	52 UACC-62	53 UO-31
Med	0.01	0.00	-0.02	0.03	0.03	0.03	-0.02	0.05	0.01	0.01	0.00	0.01	-0.02	0.00	0.01	-0.01	0.02
Mad	0.04	0.03	0.04	0.04	0.04	0.04	0.02	0.03	0.03	0.03	0.02	0.04	0.04	0.04	0.07	0.05	0.03
Mean	0.02	0.00	-0.02	0.03	0.02	0.01	-0.01	0.04	0.02	0.00	-0.00	0.01	-0.01	-0.01	0.00	0.01	0.01
Std	0.05	0.04	0.06	0.05	0.04	0.06	0.03	0.04	0.04	0.05	0.03	0.05	0.06	0.06	0.09	0.06	0.04
Min	-0.09	-0.08	-0.10	-0.06	-0.04	-0.11	-0.06	-0.03	-0.01	-0.09	-0.04	-0.07	-0.10	-0.09	-0.12	-0.06	-0.09
Max	0.09	0.08	0.08	0.09	0.07	0.06	0.07	0.10	0.10	0.06	0.04	0.08	0.08	0.08	0.15	0.11	0.06
Pct25	-0.01	-0.02	-0.05	0.02	-0.02	-0.02	-0.02	0.04	0.00	-0.00	-0.01	-0.01	-0.05	-0.04	-0.08	-0.03	0.01
Pct75	0.04	0.03	0.02	0.05	0.06	0.05	-0.01	0.07	0.04	0.02	0.01	0.02	0.03	0.02	0.06	0.04	0.03

Fig.2 is the epoch=0 Pearson cross coefficient (PCC) between real bliss score and predicted bliss score per cell line from all cross-validation iterations

PCC of Training Set The final PCC of training set (cf. Fig.2) shows that the correlation between the real and predicted bliss scores are around 0.98. It suggests that simple model is capable of producing relatively high training accuracy.

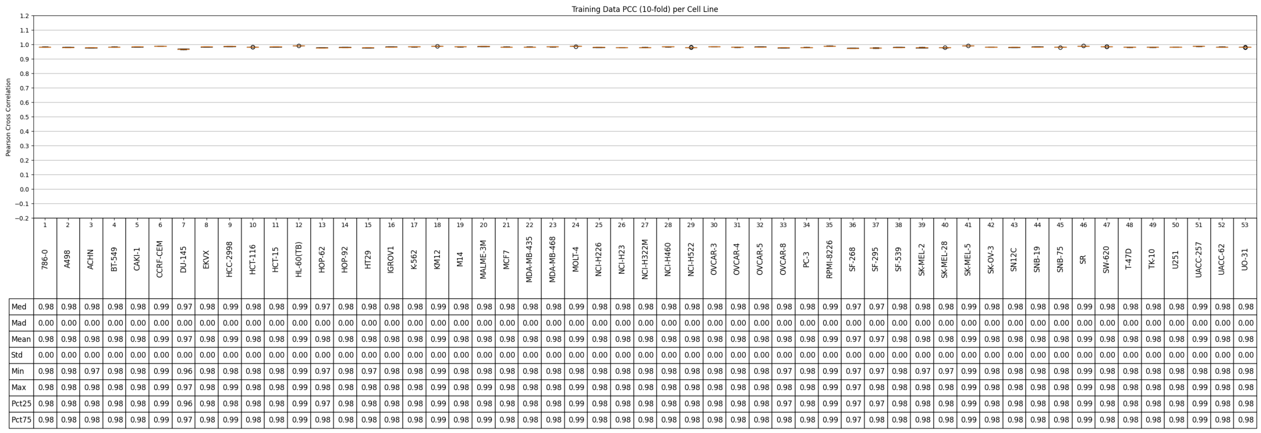

	1 786-0	2 A498	3 ACHN	4 BT-549	5 CAKI-1	6 CCRF-CEM	7 DU-145	8 EKVX	9 HCC-2998	10 HCT-116	11 HCT-15	12 HL-60(TB)	13 HOP-62	14 HOP-92	15 HT29	16 IGROV1	17 K-562	18 KM12
Med	0.98	0.98	0.98	0.98	0.98	0.99	0.97	0.98	0.99	0.98	0.98	0.99	0.97	0.98	0.98	0.98	0.98	0.99
Mad	0.00	0.00	0.00	0.00	0.00	0.00	0.00	0.00	0.00	0.00	0.00	0.00	0.00	0.00	0.00	0.00	0.00	0.00
Mean	0.98	0.98	0.98	0.98	0.98	0.99	0.97	0.98	0.99	0.98	0.98	0.99	0.98	0.98	0.98	0.98	0.98	0.99
Std	0.00	0.00	0.00	0.00	0.00	0.00	0.00	0.00	0.00	0.00	0.00	0.00	0.00	0.00	0.00	0.00	0.00	0.00
Min	0.98	0.98	0.97	0.98	0.98	0.99	0.96	0.98	0.98	0.98	0.98	0.99	0.97	0.98	0.97	0.98	0.98	0.99
Max	0.98	0.98	0.98	0.98	0.98	0.99	0.97	0.98	0.99	0.98	0.98	0.99	0.98	0.98	0.98	0.98	0.98	0.99
Pct25	0.98	0.98	0.98	0.98	0.98	0.99	0.96	0.98	0.98	0.98	0.98	0.99	0.97	0.98	0.98	0.98	0.98	0.99
Pct75	0.98	0.98	0.98	0.98	0.98	0.99	0.97	0.98	0.99	0.98	0.98	0.99	0.98	0.98	0.98	0.98	0.98	0.99

	19 M14	20 MALME-3M	21 MCF7	22 MDA-MB-435	23 MDA-MB-468	24 MOLT-4	25 NCI-H226	26 NCI-H23	27 NCI-H322M	28 NCI-H460	29 NCI-H522	30 OVCAR-3	31 OVCAR-4	32 OVCAR-5	33 OVCAR-8	34 PC-3	35 RPMI-8226	36 SF-268
Med	0.98	0.98	0.98	0.98	0.98	0.99	0.98	0.98	0.98	0.98	0.98	0.98	0.98	0.98	0.98	0.98	0.99	0.97
Mad	0.00	0.00	0.00	0.00	0.00	0.00	0.00	0.00	0.00	0.00	0.00	0.00	0.00	0.00	0.00	0.00	0.00	0.00
Mean	0.98	0.98	0.98	0.98	0.98	0.99	0.98	0.98	0.98	0.98	0.98	0.98	0.98	0.98	0.98	0.98	0.99	0.97
Std	0.00	0.00	0.00	0.00	0.00	0.00	0.00	0.00	0.00	0.00	0.00	0.00	0.00	0.00	0.00	0.00	0.00	0.00
Min	0.98	0.98	0.98	0.98	0.98	0.98	0.98	0.98	0.98	0.98	0.98	0.98	0.98	0.98	0.97	0.98	0.99	0.97
Max	0.98	0.99	0.98	0.98	0.98	0.99	0.98	0.98	0.98	0.98	0.98	0.98	0.98	0.98	0.98	0.98	0.99	0.97
Pct25	0.98	0.98	0.98	0.98	0.98	0.99	0.98	0.98	0.98	0.98	0.98	0.98	0.98	0.98	0.97	0.98	0.99	0.97
Pct75	0.98	0.99	0.98	0.98	0.98	0.99	0.98	0.98	0.98	0.98	0.98	0.98	0.98	0.98	0.98	0.98	0.99	0.97

	37 SF-295	38 SF-539	39 SK-MEL-2	40 SK-MEL-28	41 SK-MEL-5	42 SK-OV-3	43 SN12C	44 SNB-19	45 SNB-75	46 SR	47 SW-620	48 T-47D	49 TK-10	50 U251	51 UACC-257	52 UACC-62	53 UO-31
Med	0.97	0.98	0.98	0.98	0.99	0.98	0.98	0.98	0.98	0.99	0.98	0.98	0.98	0.98	0.99	0.98	0.98
Mad	0.00	0.00	0.00	0.00	0.00	0.00	0.00	0.00	0.00	0.00	0.00	0.00	0.00	0.00	0.00	0.00	0.00
Mean	0.97	0.98	0.98	0.98	0.99	0.98	0.98	0.98	0.98	0.99	0.98	0.98	0.98	0.98	0.99	0.98	0.98
Std	0.00	0.00	0.00	0.00	0.00	0.00	0.00	0.00	0.00	0.00	0.00	0.00	0.00	0.00	0.00	0.00	0.00
Min	0.97	0.98	0.97	0.97	0.99	0.98	0.98	0.98	0.98	0.99	0.98	0.98	0.98	0.98	0.98	0.98	0.98
Max	0.98	0.98	0.98	0.98	0.99	0.98	0.98	0.98	0.98	0.99	0.98	0.98	0.98	0.98	0.99	0.98	0.98
Pct25	0.97	0.98	0.98	0.98	0.99	0.98	0.98	0.98	0.98	0.99	0.98	0.98	0.98	0.98	0.99	0.98	0.98
Pct75	0.98	0.98	0.98	0.98	0.99	0.98	0.98	0.98	0.98	0.99	0.98	0.98	0.98	0.98	0.99	0.98	0.98

Fig.3 is the final training set Pearson cross coefficient (PCC) between real bliss score and predicted bliss score per cell line of from all cross-validation iterations

PCC of Validating Set The final PCC of validating set (cf. Fig.3) shows that the correlation between the real and predicted bliss scores are around 0.6 with few cell lines shows extremely high (SK-MEL-5) and low (HT29) correlation.

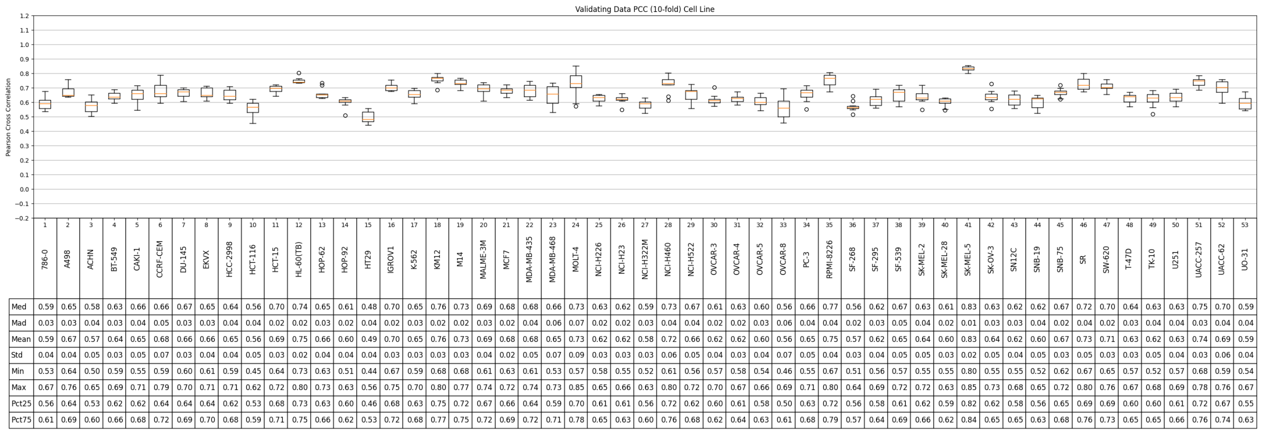

	1 786-0	2 A498	3 ACHN	4 BT-549	5 CAKI-1	6 CCRF-CEM	7 DU-145	8 EKVX	9 HCC-2998	10 HCT-116	11 HCT-15	12 HL-60(TB)	13 HOP-62	14 HOP-92	15 HT29	16 IGROV1	17 K-562	18 KM12
Med	0.59	0.65	0.58	0.63	0.66	0.66	0.67	0.65	0.64	0.56	0.70	0.74	0.65	0.61	0.48	0.70	0.65	0.76
Mad	0.03	0.03	0.04	0.03	0.04	0.05	0.03	0.03	0.04	0.04	0.02	0.02	0.03	0.02	0.04	0.02	0.03	0.02
Mean	0.59	0.67	0.57	0.64	0.65	0.68	0.66	0.66	0.65	0.56	0.69	0.75	0.66	0.60	0.49	0.70	0.65	0.76
Std	0.04	0.04	0.05	0.03	0.05	0.07	0.03	0.04	0.04	0.05	0.03	0.02	0.04	0.04	0.04	0.03	0.03	0.03
Min	0.53	0.64	0.50	0.59	0.55	0.59	0.60	0.61	0.59	0.45	0.64	0.73	0.63	0.51	0.44	0.67	0.59	0.68
Max	0.67	0.76	0.65	0.69	0.71	0.79	0.70	0.71	0.71	0.62	0.72	0.80	0.73	0.63	0.56	0.75	0.70	0.80
Pct25	0.56	0.64	0.53	0.62	0.62	0.64	0.64	0.64	0.62	0.53	0.68	0.73	0.63	0.60	0.46	0.68	0.63	0.75
Pct75	0.61	0.69	0.60	0.66	0.68	0.72	0.69	0.70	0.68	0.59	0.71	0.75	0.66	0.62	0.53	0.72	0.68	0.77

	19 M14	20 MALME-3M	21 MCF7	22 MDA-MB-435	23 MDA-MB-468	24 MOLT-4	25 NCI-H226	26 NCI-H23	27 NCI-H322M	28 NCI-H460	29 NCI-H522	30 OVCAR-3	31 OVCAR-4	32 OVCAR-5	33 OVCAR-8	34 PC-3	35 RPMI-8226	36 SF-268
Med	0.73	0.69	0.68	0.68	0.66	0.73	0.63	0.62	0.59	0.73	0.67	0.61	0.63	0.60	0.56	0.66	0.77	0.56
Mad	0.02	0.03	0.02	0.04	0.06	0.07	0.02	0.02	0.03	0.04	0.04	0.02	0.02	0.03	0.06	0.04	0.04	0.02
Mean	0.73	0.69	0.68	0.68	0.65	0.73	0.62	0.62	0.58	0.72	0.66	0.62	0.62	0.60	0.56	0.65	0.75	0.57
Std	0.03	0.04	0.02	0.05	0.07	0.09	0.03	0.03	0.03	0.06	0.05	0.04	0.03	0.04	0.07	0.05	0.04	0.03
Min	0.68	0.61	0.63	0.61	0.53	0.57	0.58	0.55	0.52	0.61	0.56	0.57	0.58	0.54	0.46	0.55	0.67	0.51
Max	0.77	0.74	0.72	0.74	0.73	0.85	0.65	0.66	0.63	0.80	0.72	0.70	0.67	0.66	0.69	0.71	0.80	0.64
Pct25	0.72	0.67	0.66	0.64	0.59	0.70	0.61	0.61	0.56	0.72	0.62	0.60	0.61	0.58	0.50	0.63	0.72	0.56
Pct75	0.75	0.72	0.69	0.72	0.71	0.78	0.65	0.63	0.60	0.76	0.68	0.62	0.64	0.63	0.61	0.68	0.79	0.57

	37 SF-295	38 SF-539	39 SK-MEL-2	40 SK-MEL-28	41 SK-MEL-5	42 SK-OV-3	43 SN12C	44 SNB-19	45 SNB-75	46 SR	47 SW-620	48 T-47D	49 TK-10	50 U251	51 UACC-257	52 UACC-62	53 UO-31
Med	0.62	0.67	0.63	0.61	0.83	0.63	0.62	0.62	0.67	0.72	0.70	0.64	0.63	0.63	0.75	0.70	0.59
Mad	0.03	0.05	0.04	0.02	0.01	0.03	0.03	0.04	0.02	0.04	0.02	0.03	0.04	0.04	0.03	0.04	0.04
Mean	0.62	0.65	0.64	0.60	0.83	0.64	0.62	0.60	0.67	0.73	0.71	0.63	0.62	0.63	0.74	0.69	0.59
Std	0.04	0.05	0.05	0.03	0.02	0.05	0.04	0.05	0.03	0.05	0.03	0.03	0.05	0.04	0.03	0.06	0.04
Min	0.56	0.57	0.55	0.55	0.80	0.55	0.55	0.52	0.62	0.67	0.65	0.57	0.52	0.57	0.68	0.59	0.54
Max	0.69	0.72	0.72	0.63	0.85	0.73	0.68	0.65	0.72	0.80	0.76	0.67	0.68	0.69	0.78	0.76	0.67
Pct25	0.58	0.61	0.62	0.59	0.82	0.62	0.58	0.56	0.65	0.69	0.69	0.60	0.60	0.61	0.72	0.67	0.55
Pct75	0.64	0.69	0.66	0.62	0.84	0.65	0.65	0.63	0.68	0.76	0.73	0.65	0.65	0.66	0.76	0.74	0.63

Fig.4 is the final validating set Pearson cross coefficient (PCC) between real bliss score and predicted bliss score per cell line at epoch=2048 of from all cross-validation iterations

S2.2 Hyperparameter Tuning of Algorithms

The goal of hyperparameter tuning is to confirm the global optimal choice of parameters for SynAI DL model. Moreover, an additional choices of popular regression solutions were included as a reference/comparison to SynAI performance. The following regression solutions were tested and compared to SynAI. The selection is based on existing literature on drug synergism prediction study (An2022). Here we employ out-of-box toolbox library (**scikit-learn**) to provide the other regressor implementations. The final test PCC (Pearson cross correlation) results are elobrated at Table. 1. The reason of using PCC as the algorithm performance criteria is based on existing literature study (An2022) as different algorithm may employ different loss function during their fitting/training procedure which makes the algorithm comparison impossible. However, PCC caculation between measured and predicted synergy reading is independent of algorithm and strongly correlated to loss function such as mean-square-error (MSE) or mean-absolut-error (MAE). In addition, PCC value range is scale-free and independent from data variation.

- Random forset regression (RandomForestRegressor from scikit-learn library)
- Gradient boosted regression (GradientBoostingRegressor from scikit-learn library)
- Recurrent neural network (RNN from pytorch library)

To ensure an objective comparison crossing regressor models, a hyperparameter tuning was performed using grid search strategy provided by scikit-learn library. Search over specified parameter values with successive halving. The search strategy starts evaluating all the candidates with a small amount of resources and iteratively selects the best candidates, using more and more resources.

In conclusion (cf. Table.1), compared to SynAI, RNN model is able to produce a very similar yet lower performance to SynAI. Random foreset (RF) and Gradient Boosting (GBX) regression solutions both yield lower performance than SynAI. Inital test of all algorithms show tendency of overfitting using the default parameter setting, thus here we deliberately introduce higher dropout and lowering the complexity of the model by reducing key parameter contributing to algorithm complexity. For example, in RF algorithm we use much higher number of estimator while lower maximum tree depth to avoid overfitting. Similarity for GBX, much lower value of minimum sample leaf and minimum sample split are employed during the tuning test. The same goes for RNN and SynAI as well with much smaller number of hidden layer and hidden layer sizes are employed.

The hyperparameters for each regressor algorithms are elobrated in the following sections. Based on the tuning tests, it is difficult to observe an unanimous choice of hyperparameters crossing different cell line for each algorithms. The hypothesis is that each cell model possesses a different genetic profile which may response differently to the same combination of drugs. In addition, the tuning tests confirms that a single prediction model scenario may not be the optimal solution. Intead, a per-cell model training strategy would yield higher performance. This conclusion is also in-line with previous literature such as Holbeck 2017 and Liu 2016.

Table.1 comparison of regression solutions crossing three cell line from NCI-Almanac dataset

Algorithm	MCF7	OVCAR-8	SK-MEL-5
SynAI	0.68 ± 0.02	0.56 ± 0.07	0.86 ± 0.02
RF	0.64 ± 0.02	0.55 ± 0.03	0.89 ± 0.02
GBX	0.66 ± 0.02	0.48 ± 0.05	0.88 ± 0.02
RNN	0.54 ± 0.12	0.43 ± 0.06	0.83 ± 0.08

S2.2.1 hyperparameter for SynAI regressor

The result of HalveGridSearching shows that the final test set PCC are converging for each cell lines. In total, a full combination of 288 parameter sets were tested at iteration 0 and it narrows down to a smaller (49) collection of parameter sets (cf. Fig.4 ~ 6) in the iteration 2 (final). However, there is no clear indication of a global optimal parameter set (cf. Fig.6).

- **net_choice** is the choice of the DL network
- **loss_choice** is the choice of loss function defined by pytorch framework, popular choices including MSE (mean squared error) and MAE (mean absolute error)
- **optim_choice** is the choice of optimizer function defined by pytorch framework, popular choices includings
  - **SGD**: implements stochastic gradient descent (optionally with momentum).
  - **ASGD**: implements Averaged Stochastic Gradient Descent (SGD)
  - **Adam**: an algorithm for first-order gradient-based optimization of stochastic objective functions, based on adaptive estimates of lower-order moments
  - **RAdam**: this implementation uses the same weight_decay implementation as Adam (were the weight_decay is applied to the gradient)

Code Reference

```
# hyperparameter for SynAI AI regressor
param_grid = {
    "net_choice": ["L1", "L2", "L4"],  # number of hidden layer in the perceptron
network
    "loss_choice": ['MSE'],  # choice of loss function
    "optim_choice": ['RAdam', 'SGD'],  # choice of optimizer
    "amp": [2, 1, 0.5, 0.25],  # choice of hidden layer size, in percentage of number
of dimension of input data
    "dropout": [0.0, 0.2, 0.4, 0.6],  # dropout layer percentage
    "lr": [1e-3, 1e-4, 1e-5]  # learning rate for network
}
...
sh = HalvingGridSearchCV(
    estimator=base_estimator,
    param_grid=param_grid,
    cv=5,  # cross-validation 5-folding
    verbose=100,  # verbose output
    return_train_score=True,  # make sure training score is also captured
    min_resources=256,
    n_jobs=1
).fit(all_fps, all_scores)

...
```

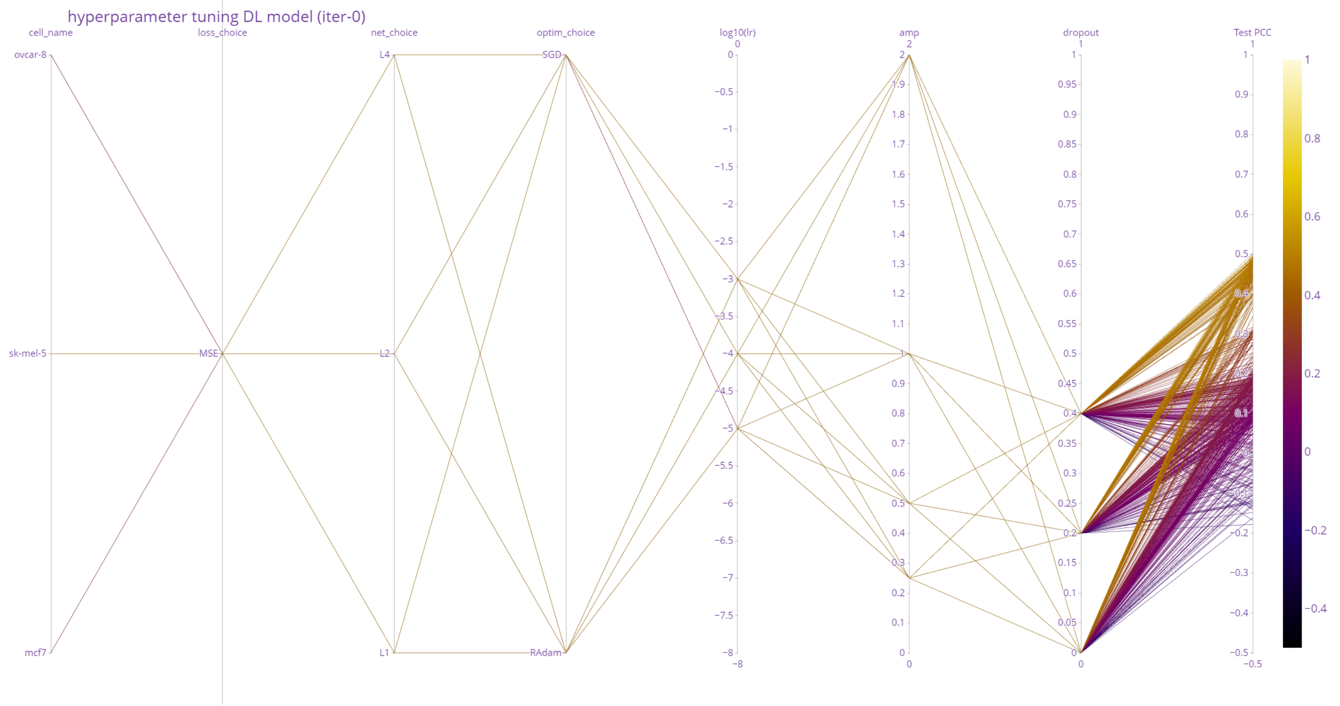

Fig 5. hyperparameter tuning results using HalveGridSearching at iter-0. The test starts with a full permutation of 288 possible combination of SynAI hyperparameter set. The results (test set PCC) are dispersing over choices of hyperparameter combinations.

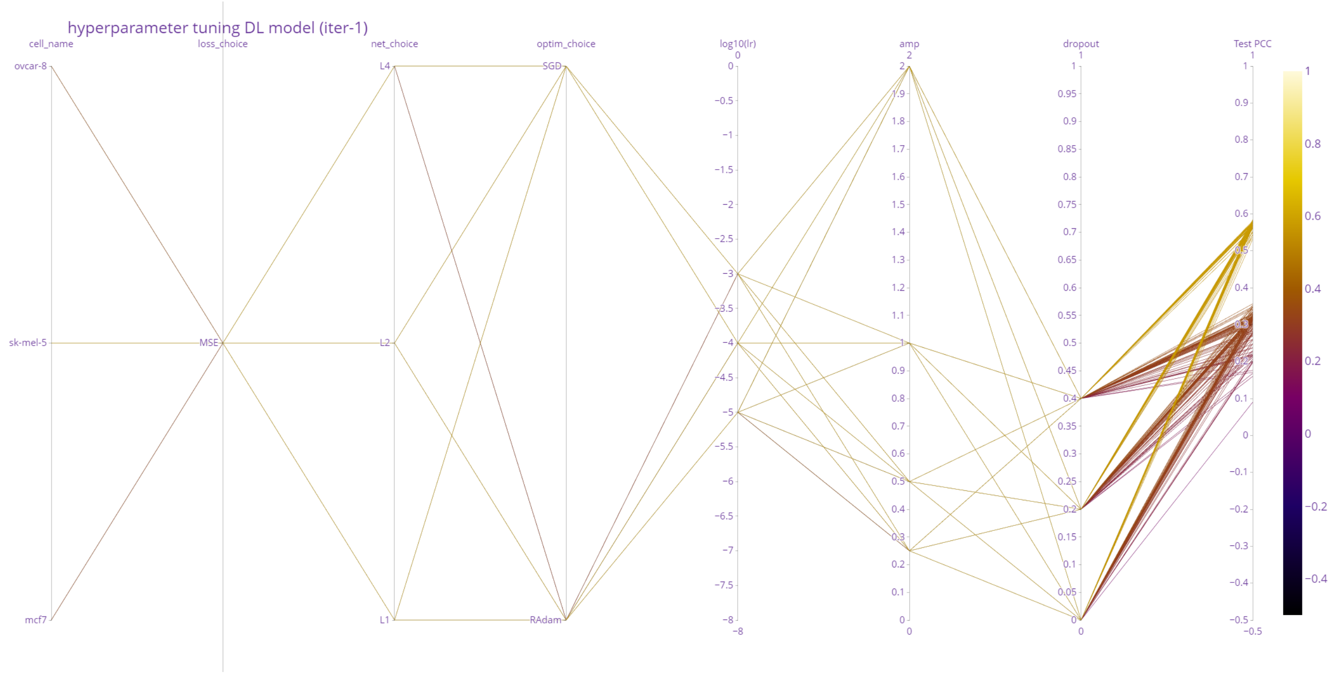

Fig 6. hyperparameter tuning results using HalveGridSearching at iter-1. The test set PCC starts to converge and different combination of hyperparameters are producing similar performance in test set.

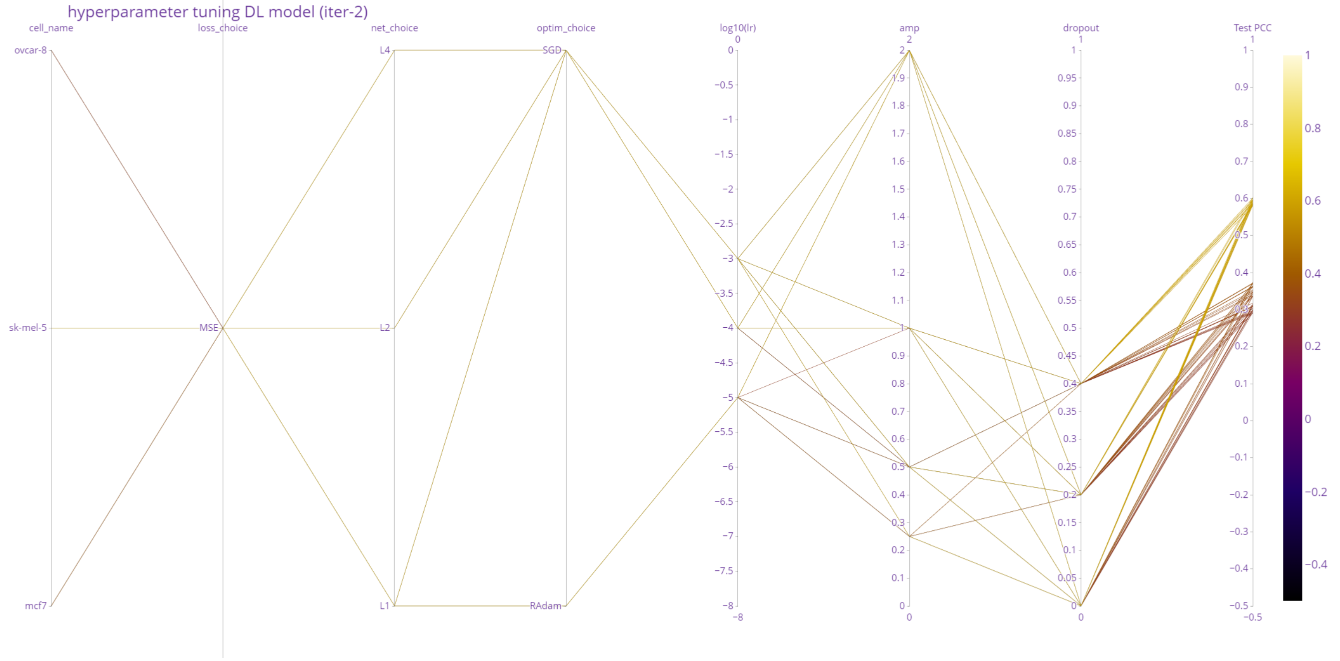

Fig 7. hyperparameter tuning results using HalveGridSearching at iter-2. The test set PCC are converged and the final hyperparameters are producing nearly identical performance.

S2.2.2 hyperparameter for RNN regressor

RNN is a popular choice of DL model simlar to multi-layer perceptron (MLP) network employed by SynAI. RNN and MLP are both popualr choices for classification and regression problem. Although CNN is often considered as well, it is more popular in the image domain and sequence domain. Here the compound is first transfered into molecular fingerprint which is a standard tabular data rather than sequence data. Here we employ default implementation of RNN provided by PyTorch library.

```
# Partial code reference

...
# definiton of regressor
    self.layer_rnn = nn.RNN(
        input_size=input_size,
        hidden_size=hidden_size,
        num_layers=num_layers,
        bias=bias,
        dropout=dropout
    )
    self.layer_fc = nn.Linear(hidden_size, 1)


def forward(self, x):
    out, hn = self.layer_rnn(x)  # RNN layer
    out = self.layer_fc(out)  # readout layer
    return out

...

<html><p style="page-break-after:always;"></p></html>

...
# hyperparameter space for RNN regressor
param_grid = {
    "net_choice": ["rnn"],
    "loss_choice": ['MSE'],
    "optim_choice": ['RAdam', 'SGD'],
    "amp": [0.5, 0.25, 0.1],
    "num_layers": [1, 2, 4],
    "dropout": [0.0, 0.2, 0.4, 0.6],
    "lr": [1e-4, 1e-5, 1e-6]
}
...

<html><p style="page-break-after:always;"></p></html>

...
# running of search

sh = HalvingGridSearchCV(
    estimator=base_estimator,
    param_grid=param_grid,
    cv=5,  # cross-validation 5-folding
    verbose=100,  # verbose output
    return_train_score=True,  # make sure training score is also captured
    min_resources=256,
```

```
    n_jobs=1
).fit(all_fps, all_scores)

...
```

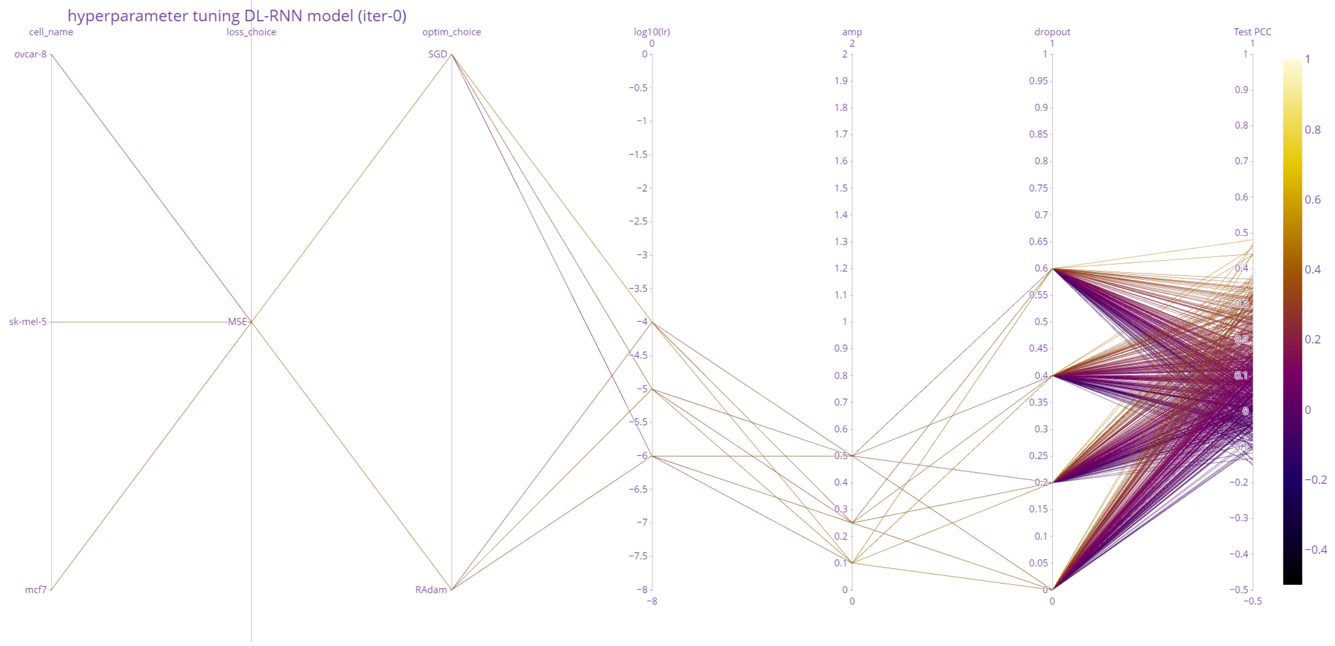

Fig 8. hyperparameter tuning results using HalveGridSearching at iter-0. The test starts with a full permutation of 216 possible combination of hyperparameter set for RNN network. The results (test set PCC) are dispersing over choices of hyperparameter combinations.

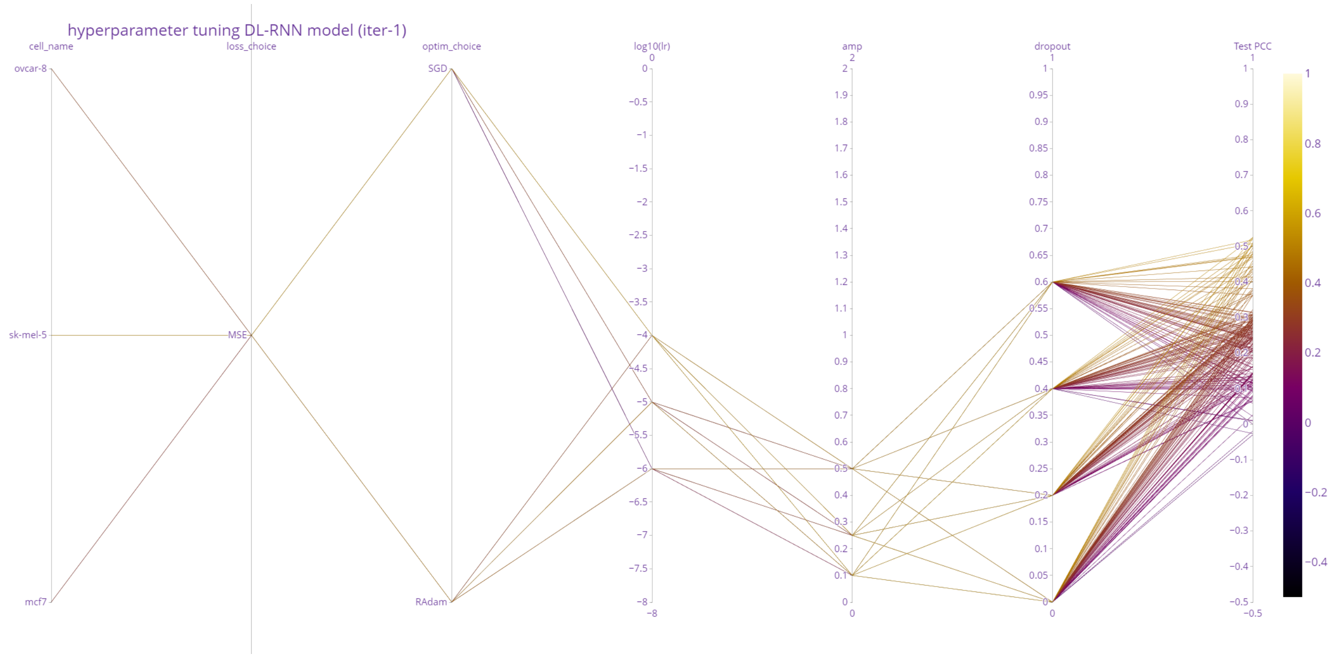

Fig 9. hyperparameter tuning results using HalveGridSearching at iter-1. The test set PCC starts to converge and different combination of hyperparameters are producing similar performance in test set.

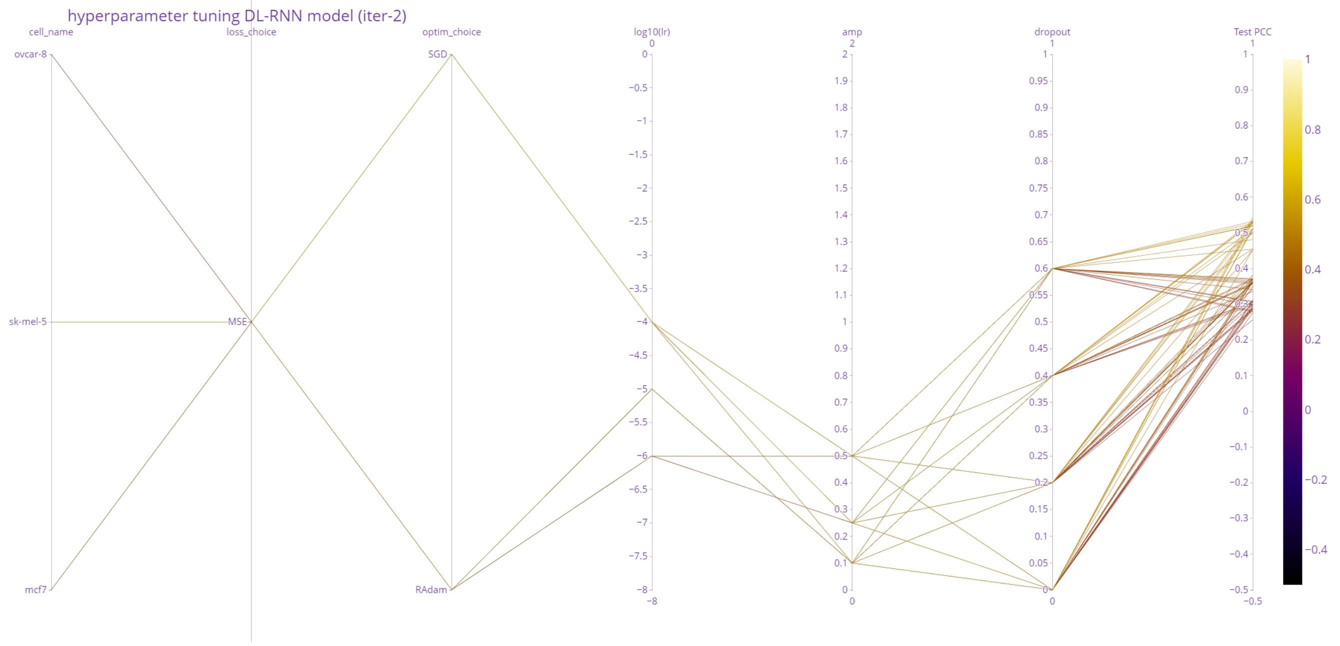

Fig 10. hyperparameter tuning results using HalveGridSearching at iter-2. The test set PCC are converged and the final hyperparameters are producing nearly identical performance. The final performance of RNN model is slightly lower than SynAI model and less converged.

S2.2.3 hyperparameter for random forest regressor

```
# Partial code reference

...
# hyperparameter search space for random forest regressor
param_grid = {
    "max_depth": [32, 256, 2048],  # key parameter, the maximum depth of the tree
    "max_leaf_nodes": [32, 256, 2048],  # key parameter, grow trees with
max_leaf_nodes in best-first fashion
    "n_estimators": [32, 256, 1024],  # key parameter, the number of trees in the
forest
    'min_samples_split': [0.05, 0.1, 0.2, 0.3],  # the minimum number of samples
required to split an internal node
    "min_samples_leaf": [0.05, 0.1, 0.2, 0.3]  # the minimum number of samples
required to be at a leaf node
}
...


...
# definition of regressor
base_estimator = RandomForestRegressor(random_state=0)

# running of search
sh = HalvingGridSearchCV(
    estimator=base_estimator,
    param_grid=param_grid,
    cv=5,
    verbose=100,
    factor=2,
    min_resources=256,
    n_jobs=6
).fit(all_fps, all_scores.ravel())
...
```

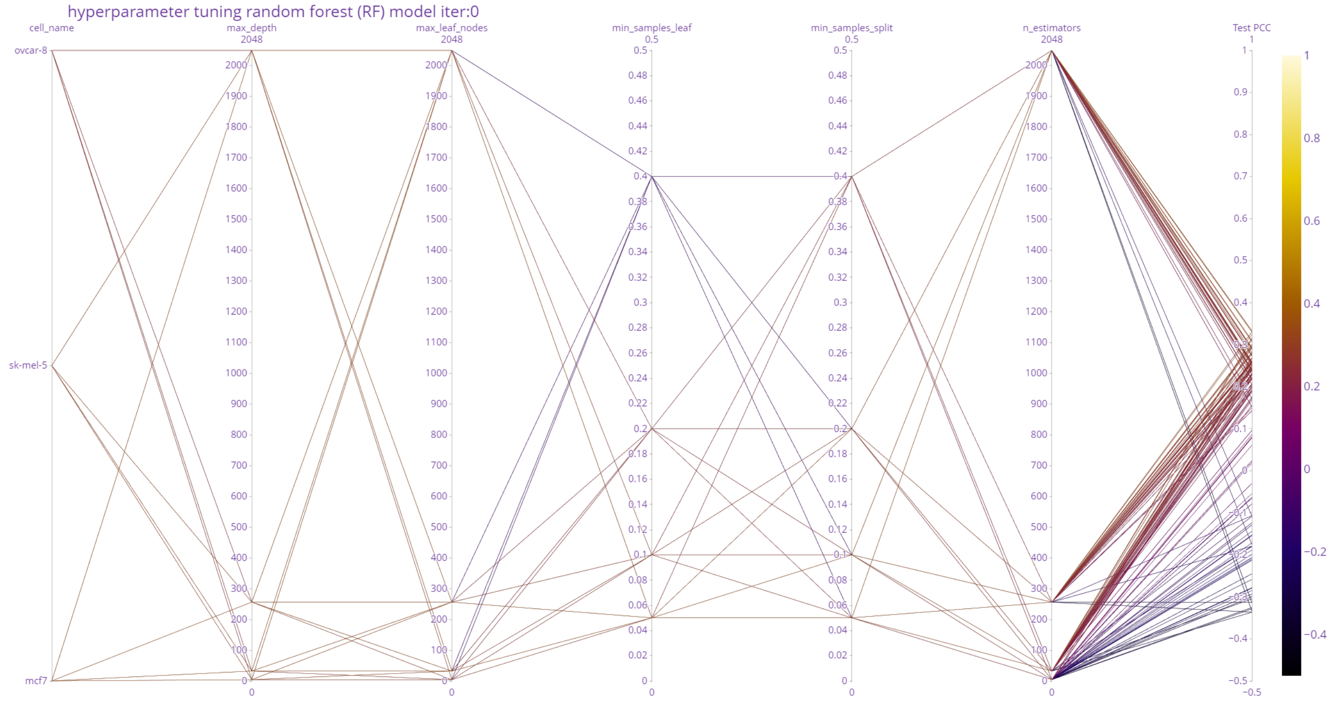

Fig 11. hyperparameter tuning results of random forset regressor using HalveGridSearching at iter-0, the searching starts with a full combination of 432 parameters for three different cell lines. The PCC between real and predicted bliss score is used as the final performance criteria of the search.

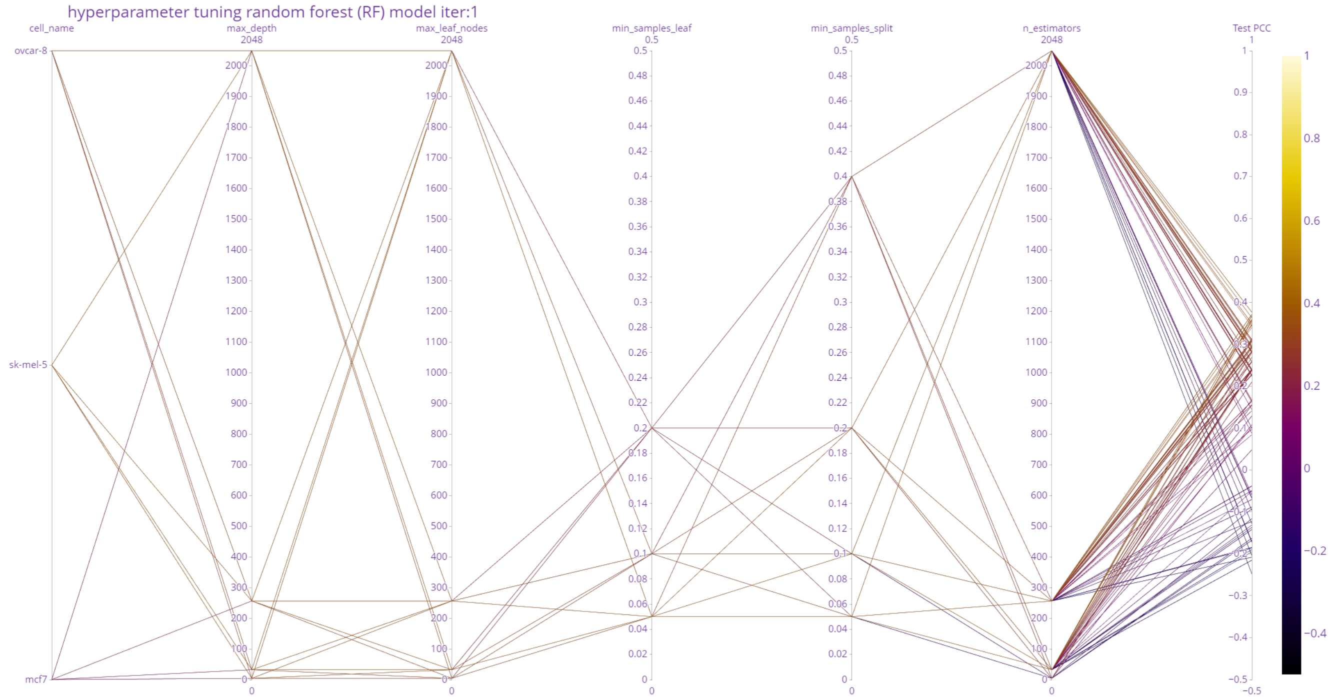

Fig 12. Iter-1 HT results of random forest regressor showing improved convergence compared to iter-0

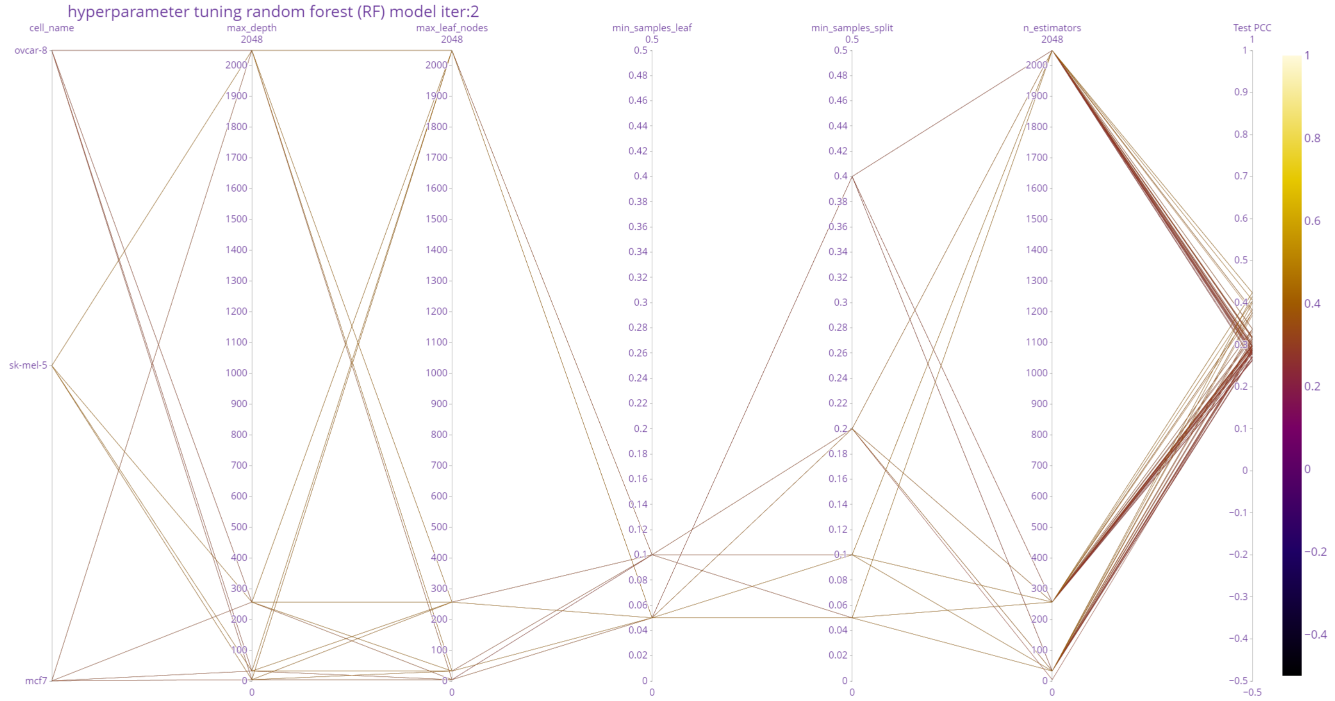

Fig 13. hyperparameter tuning results of random forset regressor using HalveGridSearching at iter-2 showing convergent result but significantly lower performance compared to either SynAI or RNN model

S2.2.4 hyperparameter for graident boosting regressor

```
# Partial code reference

...
# hyperparameter for graident boosting regressor
param_grid = {
    "max_depth": [4, 32, 256, 2048],  # the maximum depth of the tree
    "max_leaf_nodes": [4, 32, 256, 2048],  # grow trees with max_leaf_nodes in best-first fashion
    "n_estimators": [4, 32, 256, 2048],  # the number of trees in the forest
    'min_samples_split': [0.05, 0.1, 0.2, 0.4],  # the minimum number of samples required to split an internal node
    "min_samples_leaf": [0.05, 0.1, 0.2, 0.4]  # the minimum number of samples required to be at a leaf node
}
...


...
# definition of regressor
base_estimator = GradientBoostingRegressor(random_state=0)

# running of search
sh = HalvingGridSearchCV(
    estimator=base_estimator,
    param_grid=param_grid,
    cv=5,
    verbose=100,
    factor=2,
    min_resources=256,
    n_jobs=6
).fit(all_fps, all_scores.ravel())
...
```

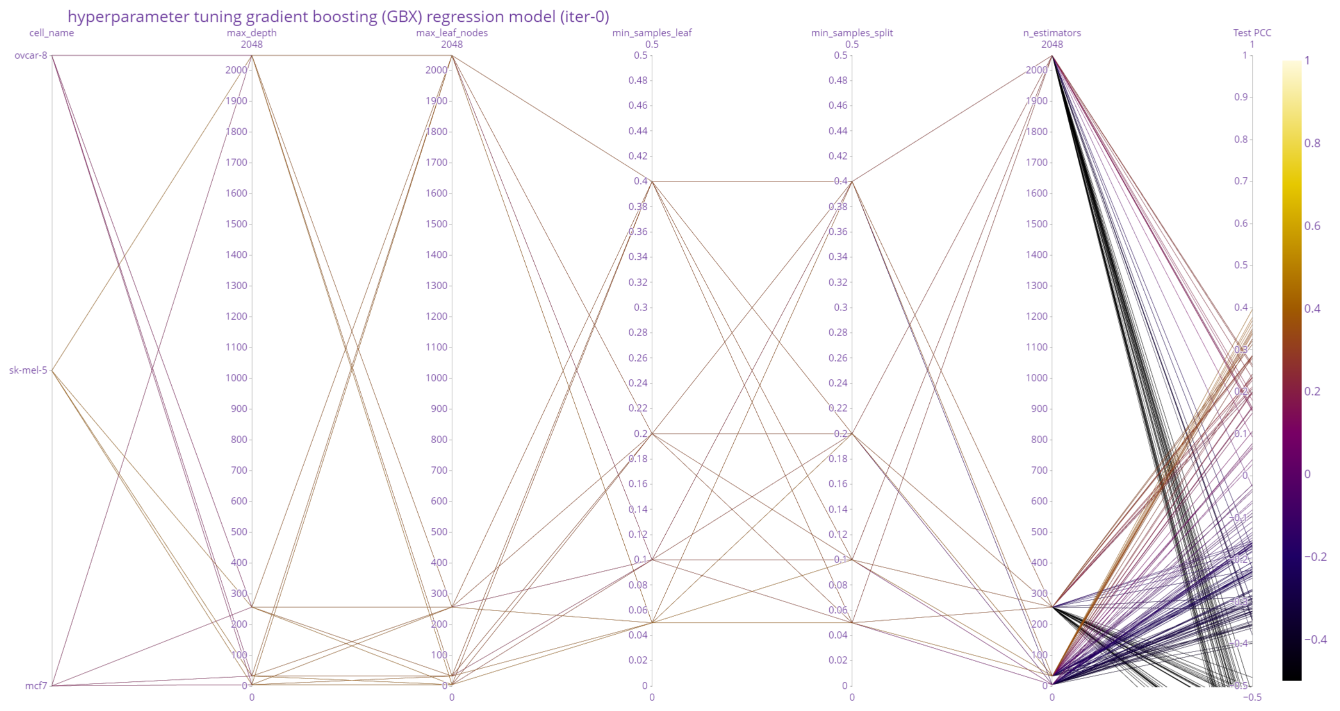

Fig 14. hyperparameter tuning results of gradient boosting regressor using HalveGridSearching at iter-0. No clear convergent results can be observed. Results of training is dispersing

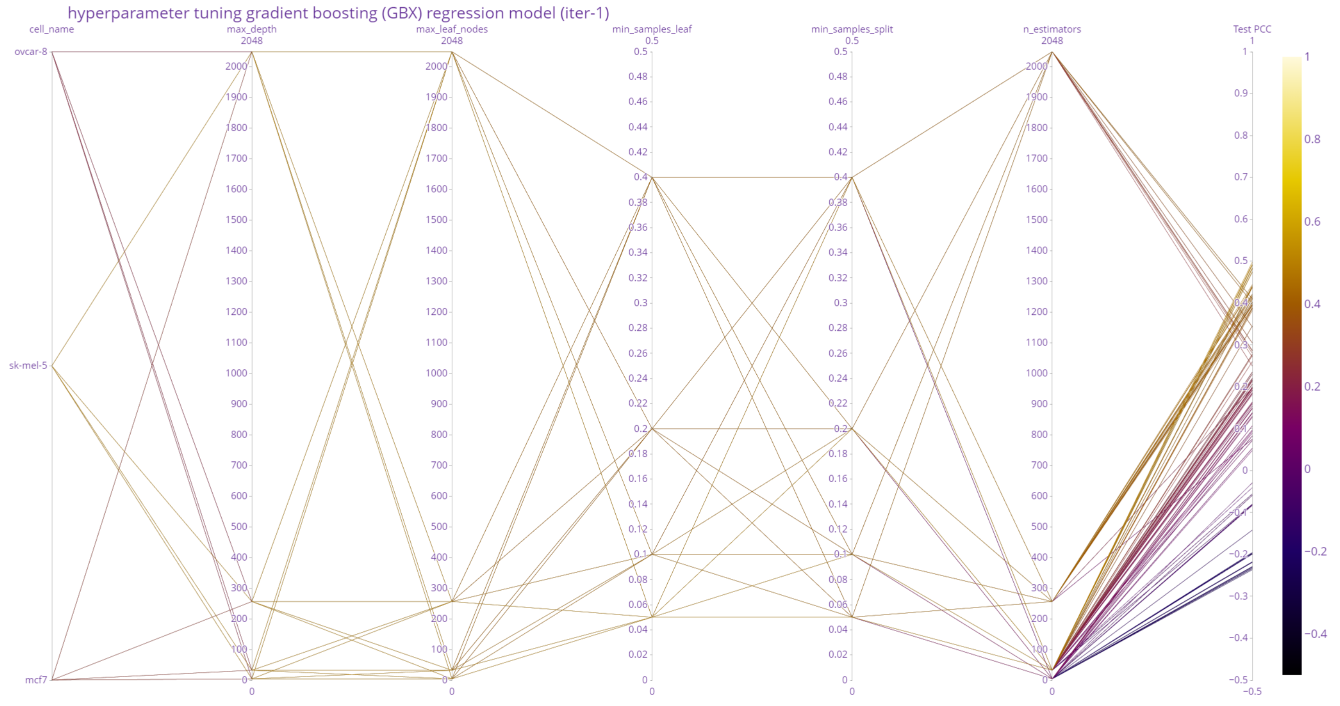

Fig 15. hyperparameter tuning results of gradient boosting regressor using HalveGridSearching at iter-1

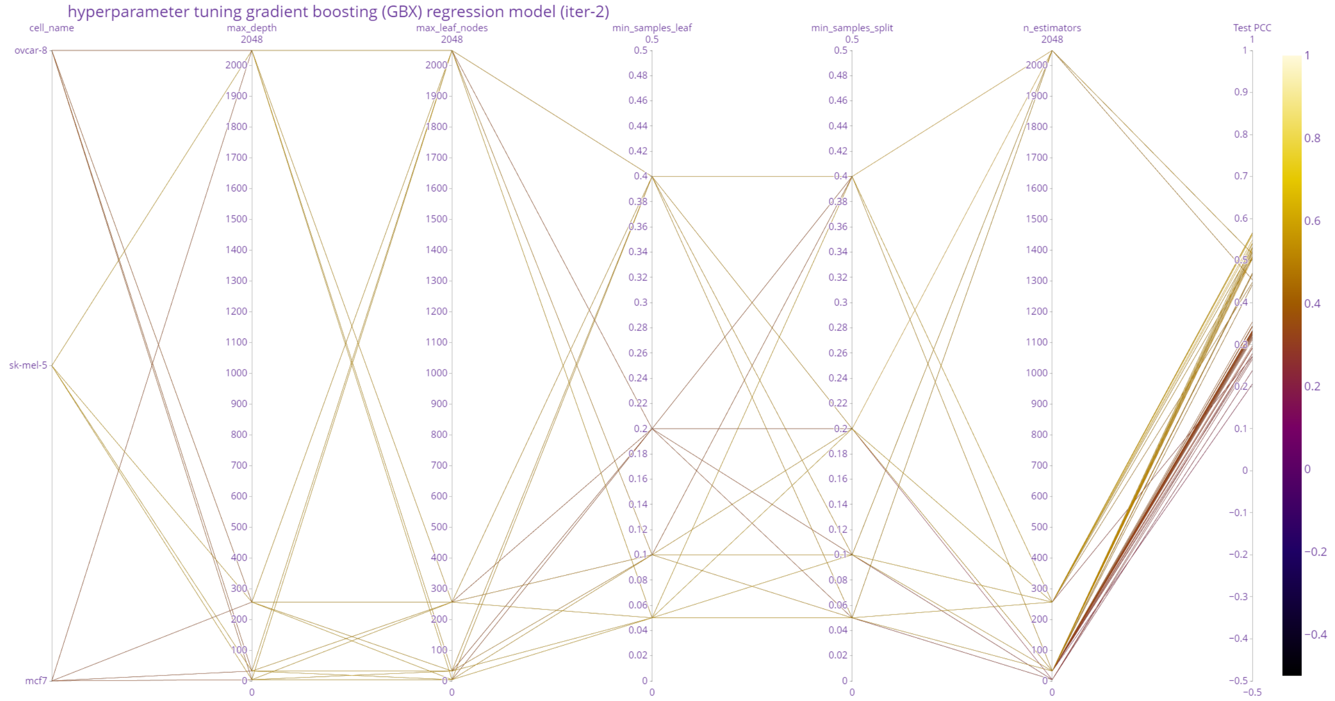

Fig 16. hyperparameter tuning results of graident boostring regressor using HalveGridSearching at iter-2 showing convergent result but significantly lower performance compared to either SynAI or RNN model. The iteration 0 and 1 results show that GB regressor fitting procedure often resulting in over-fitting

S2.3 study of alternative training routine

During early study, often trained models demonstrate a tendency of overfitting. Thus, we have experienced several alternative training routine to improve general performance of the DL network. One testing routine is to combine multiple data sources to increase model generality. Two individual tests were performed:

1. training per-cell model using data combined from multiple data sources for example combining NCI and DrugCombDB datasets
2. training a single (global) model using data combined from multiple cell lines of the same datasets.

S2.3.1 training per-cell model with NCI + DrugCombDB combined

In the initial design of SynAI, the deep learning networks were designed to be retrainable with new data. The goal is allowing internal models to be updated with future data. Questions were raisen regarding which strategy is better when (1) networks are trained with all data combined (2) networks are trained with one initial dataset and retrained recursively with additional datasets. Here we run a short comparison regarding these two strategy.

1. In the retraining stategy, per cell networks were trained first using NCI dataset with the standard training workflow. The trained networks were retrained using DrugCombDB dataset again, allowing networks to adapt new information from DrugCombDB dataset. The retrain networks are validated using NCI and DrugCombDB again.

2. In the combined training strategy, per cell networks were trained with NCI + DrugCombDB datasets together. The combined datasets are treated as a single dataset for the training.

The comparison shows that retraining strategy is able to yield a higher final performance (cf. Fig. 19) while combined data training strategy seems producing a less desirable output (cf. Fig. 22). Our further analyses of the comparison data shows that the reason of less desirable output from combined data training is largely due to that model building is dominated by one dataset and the other datasets are showing very unstable performance (cf. Fig. 22). Our hypothesis suggested that a combined data set may requires network complex to increase as well to compensate the data complexity. However, such study is beyond the scope of this paper. At the current stage, the retraining strategy is producing reasonable results.

The interesting phenomena observed in Fig. 17 showing that initial performance of DrugCombDB is already approaching PCC=0.95 while the NCI is around PCC=0.0. This is indeed expected as the model was pretrained with NCI dataset already. The validating performance shows that retrained network provides better outcomes (cf. Fig. 19).

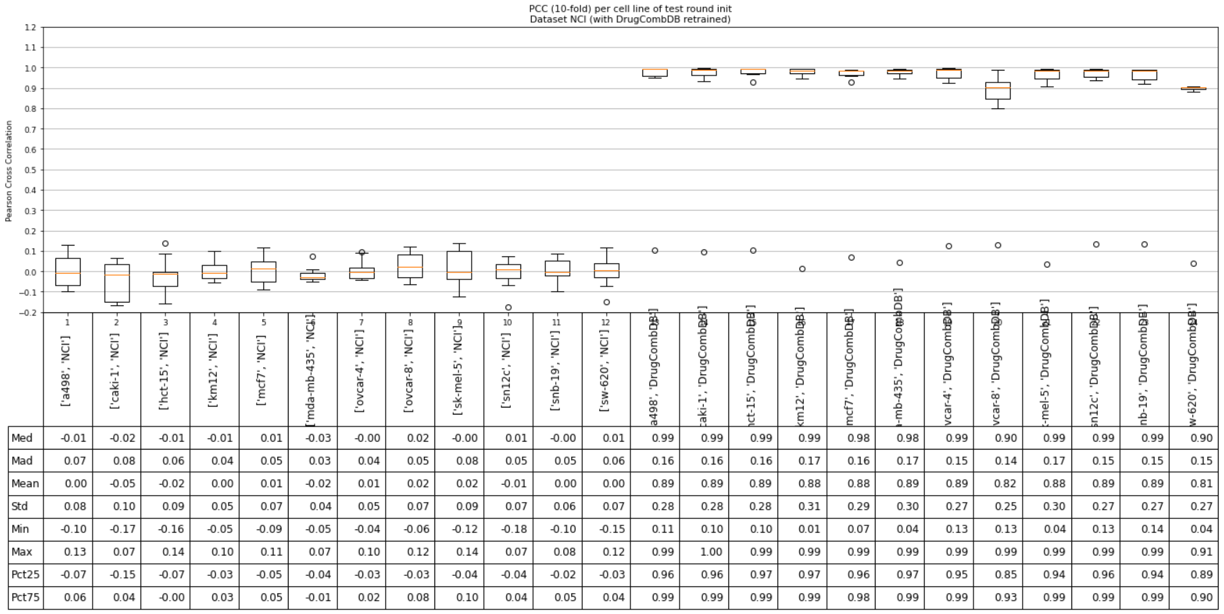

	['a498', 'NCI']	['caki-1', 'NCI']	['hct-15', 'NCI']	['km12', 'NCI']	['mcf7', 'NCI']	['mda-mb-435', 'NCI']	['ovcar-4', 'NCI']	['ovcar-8', 'NCI']	['sk-mel-5', 'NCI']	['sn12c', 'NCI']	['snb-19', 'NCI']	['sw-620', 'NCI']
Med	-0.01	-0.02	-0.01	-0.01	0.01	-0.03	-0.00	0.02	-0.00	0.01	-0.00	0.01
Mad	0.07	0.08	0.06	0.04	0.05	0.03	0.04	0.05	0.08	0.05	0.05	0.06
Mean	0.00	-0.05	-0.02	0.00	0.01	-0.02	0.01	0.02	0.02	-0.01	0.00	0.00
Std	0.08	0.10	0.09	0.05	0.07	0.04	0.05	0.07	0.09	0.07	0.06	0.07
Min	-0.10	-0.17	-0.16	-0.05	-0.09	-0.05	-0.04	-0.06	-0.12	-0.18	-0.10	-0.15
Max	0.13	0.07	0.14	0.10	0.11	0.07	0.10	0.12	0.14	0.07	0.08	0.12
Pct25	-0.07	-0.15	-0.07	-0.03	-0.05	-0.04	-0.03	-0.03	-0.04	-0.04	-0.02	-0.03
Pct75	0.06	0.04	-0.00	0.03	0.05	-0.01	0.02	0.08	0.10	0.04	0.05	0.04

	a498', 'DrugCombDB']	caki-1', 'DrugCombDB']	ct-15', 'DrugCombDB']	km12', 'DrugCombDB']	mcf7', 'DrugCombDB']	-mb-435', 'DrugCombDB']	vcar-4', 'DrugCombDB']	vcar-8', 'DrugCombDB']	-mel-5', 'DrugCombDB']	sn12c', 'DrugCombDB']	nb-19', 'DrugCombDB']	w-620', 'DrugCombDB']
Med	0.99	0.99	0.99	0.99	0.98	0.98	0.99	0.90	0.99	0.99	0.99	0.90
Mad	0.16	0.16	0.16	0.17	0.16	0.17	0.15	0.14	0.17	0.15	0.15	0.15
Mean	0.89	0.89	0.89	0.88	0.88	0.89	0.89	0.82	0.88	0.89	0.89	0.81
Std	0.28	0.28	0.28	0.31	0.29	0.30	0.27	0.25	0.30	0.27	0.27	0.27
Min	0.11	0.10	0.10	0.01	0.07	0.04	0.13	0.13	0.04	0.13	0.14	0.04
Max	0.99	1.00	0.99	0.99	0.99	0.99	0.99	0.99	0.99	0.99	0.99	0.91
Pct25	0.96	0.96	0.97	0.97	0.96	0.97	0.95	0.85	0.94	0.96	0.94	0.89
Pct75	0.99	0.99	0.99	0.99	0.98	0.99	0.99	0.93	0.99	0.99	0.99	0.90

Fig 17. Per-cell model PCC of trained with NCI and retrained with DrugCombDB for initial round

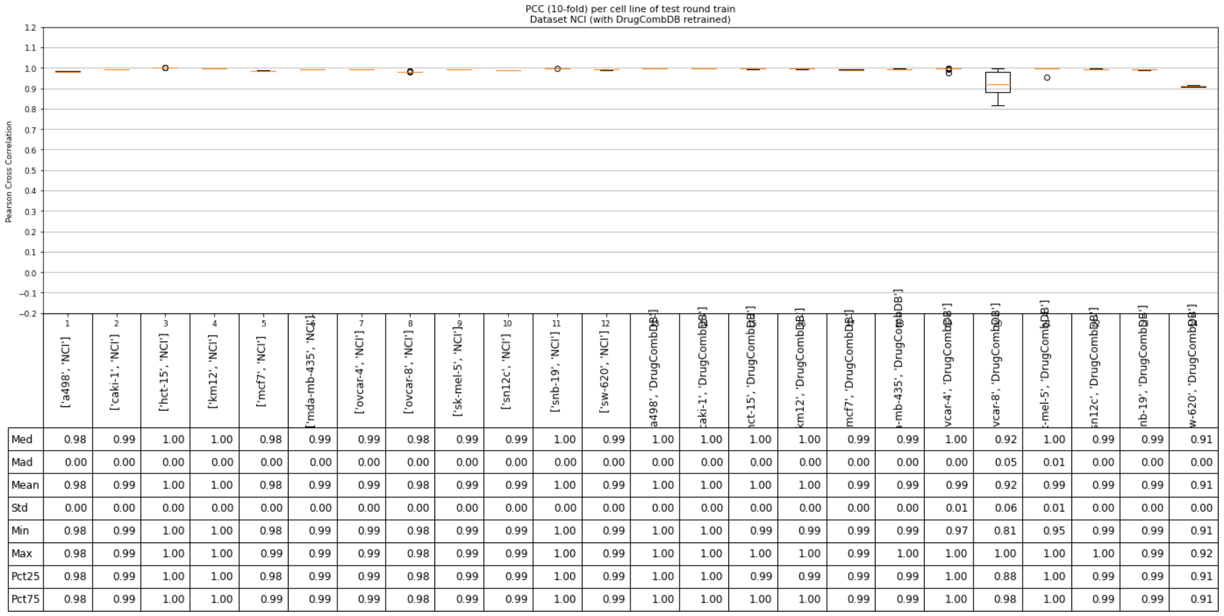

	['a498', 'NCI']	['caki-1', 'NCI']	['hct-15', 'NCI']	['km12', 'NCI']	['mcf7', 'NCI']	['mda-mb-435', 'NCI']	['ovcar-4', 'NCI']	['ovcar-8', 'NCI']	['sk-mel-5', 'NCI']	['sn12c', 'NCI']	['snb-19', 'NCI']	['sw-620', 'NCI']	a498', 'DrugCombDB']	aki-1', 'DrugCombDB']	ct-15', 'DrugCombDB']	km12', 'DrugCombDB']	mcf7', 'DrugCombDB']	-mb-435', 'DrugCombDB']	vcar-4', 'DrugCombDB']	vcar-8', 'DrugCombDB']	-mel-5', 'DrugCombDB']	sn12c', 'DrugCombDB']	nb-19', 'DrugCombDB']	w-620', 'DrugCombDB']
Med	0.98	0.99	1.00	1.00	0.98	0.99	0.99	0.98	0.99	0.99	1.00	0.99	1.00	1.00	1.00	1.00	0.99	0.99	1.00	0.92	1.00	0.99	0.99	0.91
Mad	0.00	0.00	0.00	0.00	0.00	0.00	0.00	0.00	0.00	0.00	0.00	0.00	0.00	0.00	0.00	0.00	0.00	0.00	0.00	0.05	0.01	0.00	0.00	0.00
Mean	0.98	0.99	1.00	1.00	0.98	0.99	0.99	0.98	0.99	0.99	1.00	0.99	1.00	1.00	1.00	1.00	0.99	0.99	0.99	0.92	0.99	0.99	0.99	0.91
Std	0.00	0.00	0.00	0.00	0.00	0.00	0.00	0.00	0.00	0.00	0.00	0.00	0.00	0.00	0.00	0.00	0.00	0.00	0.01	0.06	0.01	0.00	0.00	0.00
Min	0.98	0.99	1.00	1.00	0.98	0.99	0.99	0.98	0.99	0.99	1.00	0.99	1.00	1.00	0.99	0.99	0.99	0.99	0.97	0.81	0.95	0.99	0.99	0.91
Max	0.98	0.99	1.00	1.00	0.99	0.99	0.99	0.98	0.99	0.99	1.00	0.99	1.00	1.00	1.00	1.00	0.99	1.00	1.00	1.00	1.00	1.00	0.99	0.92
Pct25	0.98	0.99	1.00	1.00	0.98	0.99	0.99	0.98	0.99	0.99	1.00	0.99	1.00	1.00	0.99	0.99	0.99	0.99	1.00	0.88	1.00	0.99	0.99	0.91
Pct75	0.98	0.99	1.00	1.00	0.99	0.99	0.99	0.98	0.99	0.99	1.00	0.99	1.00	1.00	1.00	1.00	0.99	0.99	1.00	0.98	1.00	0.99	0.99	0.91

Fig 18. Per-cell model PCC of trained with NCI and retrained with DrugCombDB for training round

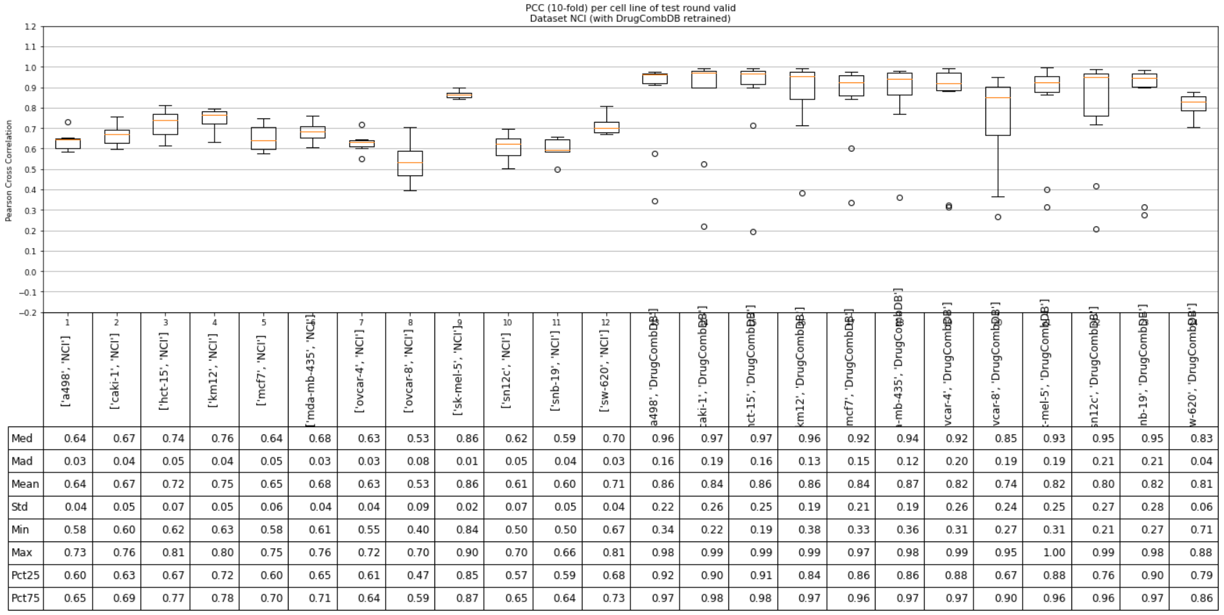

	['a498', 'NCI']	['caki-1', 'NCI']	['hct-15', 'NCI']	['km12', 'NCI']	['mcf7', 'NCI']	['mda-mb-435', 'NCI']	['ovcar-4', 'NCI']	['ovcar-8', 'NCI']	['sk-mel-5', 'NCI']	['sn12c', 'NCI']	['snb-19', 'NCI']	['sw-620', 'NCI']	a498', 'DrugCombDB']	aki-1', 'DrugCombDB']	ct-15', 'DrugCombDB']	km12', 'DrugCombDB']	mcf7', 'DrugCombDB']	-mb-435', 'DrugCombDB']	vcar-4', 'DrugCombDB']	vcar-8', 'DrugCombDB']	-mel-5', 'DrugCombDB']	sn12c', 'DrugCombDB']	nb-19', 'DrugCombDB']	w-620', 'DrugCombDB']
Med	0.64	0.67	0.74	0.76	0.64	0.68	0.63	0.53	0.86	0.62	0.59	0.70	0.96	0.97	0.97	0.96	0.92	0.94	0.92	0.85	0.93	0.95	0.95	0.83
Mad	0.03	0.04	0.05	0.04	0.05	0.03	0.03	0.08	0.01	0.05	0.04	0.03	0.16	0.19	0.16	0.13	0.15	0.12	0.20	0.19	0.19	0.21	0.21	0.04
Mean	0.64	0.67	0.72	0.75	0.65	0.68	0.63	0.53	0.86	0.61	0.60	0.71	0.86	0.84	0.86	0.86	0.84	0.87	0.82	0.74	0.82	0.80	0.82	0.81
Std	0.04	0.05	0.07	0.05	0.06	0.04	0.04	0.09	0.02	0.07	0.05	0.04	0.22	0.26	0.25	0.19	0.21	0.19	0.26	0.24	0.25	0.27	0.28	0.06
Min	0.58	0.60	0.62	0.63	0.58	0.61	0.55	0.40	0.84	0.50	0.50	0.67	0.34	0.22	0.19	0.38	0.33	0.36	0.31	0.27	0.31	0.21	0.27	0.71
Max	0.73	0.76	0.81	0.80	0.75	0.76	0.72	0.70	0.90	0.70	0.66	0.81	0.98	0.99	0.99	0.99	0.97	0.98	0.99	0.95	1.00	0.99	0.98	0.88
Pct25	0.60	0.63	0.67	0.72	0.60	0.65	0.61	0.47	0.85	0.57	0.59	0.68	0.92	0.90	0.91	0.84	0.86	0.86	0.88	0.67	0.88	0.76	0.90	0.79
Pct75	0.65	0.69	0.77	0.78	0.70	0.71	0.64	0.59	0.87	0.65	0.64	0.73	0.97	0.98	0.98	0.97	0.96	0.97	0.97	0.90	0.96	0.96	0.97	0.86

Fig 19. Per-cell model PCC of trained with NCI and retrained with DrugCombDB for validating round.

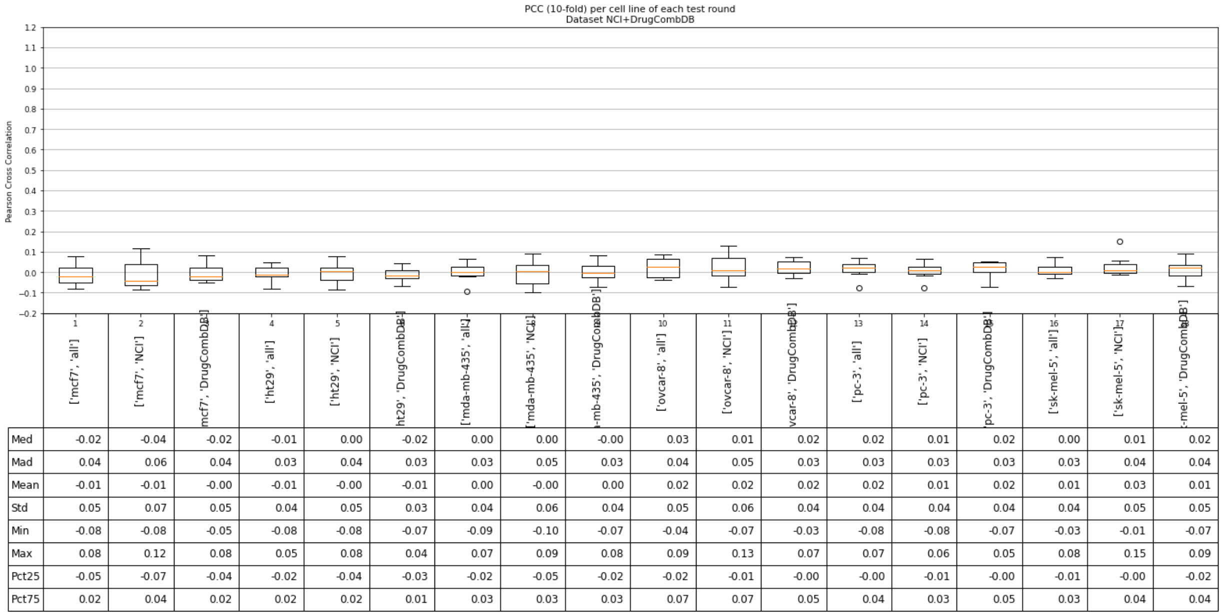

	['mcf7', 'all']	['mcf7', 'NCI']	mcf7', 'DrugCombDB']	['ht29', 'all']	['ht29', 'NCI']	ht29', 'DrugCombDB']	['mda-mb-435', 'all']	['mda-mb-435', 'NCI']	-mb-435', 'DrugCombDB']	['ovcar-8', 'all']	['ovcar-8', 'NCI']	vcar-8', 'DrugCombDB']	['pc-3', 'all']	['pc-3', 'NCI']	pc-3', 'DrugCombDB']	['sk-mel-5', 'all']	['sk-mel-5', 'NCI']	-mel-5', 'DrugCombDB']
Med	-0.02	-0.04	-0.02	-0.01	0.00	-0.02	0.00	0.00	-0.00	0.03	0.01	0.02	0.02	0.01	0.02	0.00	0.01	0.02
Mad	0.04	0.06	0.04	0.03	0.04	0.03	0.03	0.05	0.03	0.04	0.05	0.03	0.03	0.03	0.03	0.03	0.04	0.04
Mean	-0.01	-0.01	-0.00	-0.01	-0.00	-0.01	0.00	-0.00	0.00	0.02	0.02	0.02	0.02	0.01	0.02	0.01	0.03	0.01
Std	0.05	0.07	0.05	0.04	0.05	0.03	0.04	0.06	0.04	0.05	0.06	0.04	0.04	0.04	0.04	0.04	0.05	0.05
Min	-0.08	-0.08	-0.05	-0.08	-0.08	-0.07	-0.09	-0.10	-0.07	-0.04	-0.07	-0.03	-0.08	-0.08	-0.07	-0.03	-0.01	-0.07
Max	0.08	0.12	0.08	0.05	0.08	0.04	0.07	0.09	0.08	0.09	0.13	0.07	0.07	0.06	0.05	0.08	0.15	0.09
Pct25	-0.05	-0.07	-0.04	-0.02	-0.04	-0.03	-0.02	-0.05	-0.02	-0.02	-0.01	-0.00	-0.00	-0.01	-0.00	-0.01	-0.00	-0.02
Pct75	0.02	0.04	0.02	0.02	0.02	0.01	0.03	0.03	0.03	0.07	0.07	0.05	0.04	0.03	0.05	0.03	0.04	0.04

Fig 20. Per-cell model PCC of NCI+DrugCombDB dataset for initial round

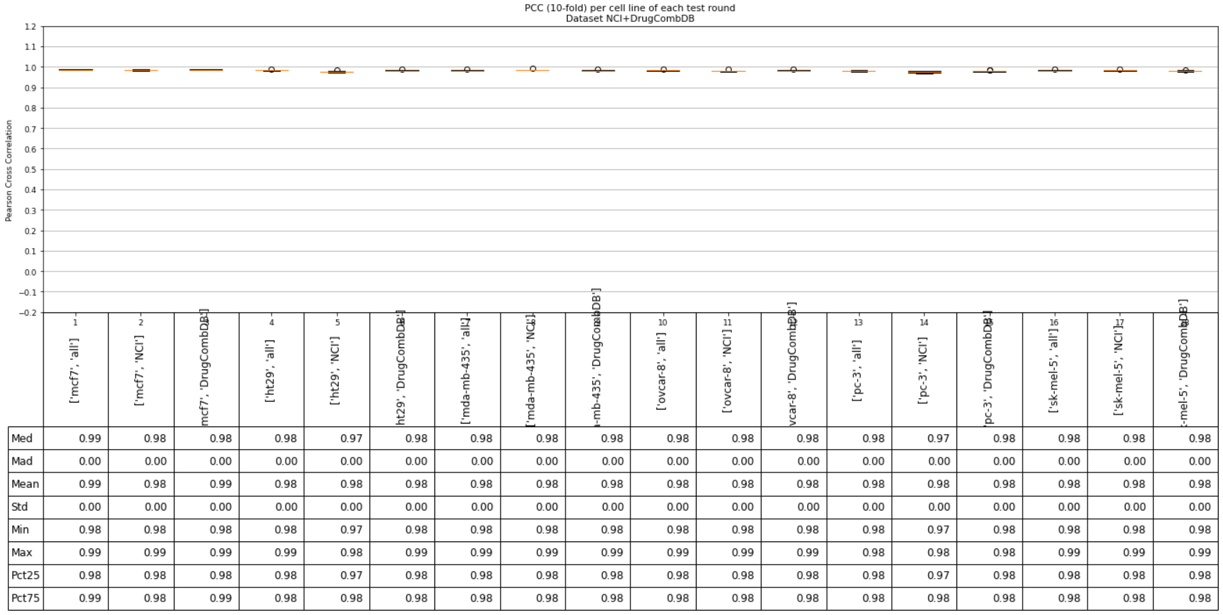

	['mcf7', 'all']	['mcf7', 'NCI']	mcf7', 'DrugCombDB']	['ht29', 'all']	['ht29', 'NCI']	ht29', 'DrugCombDB']	['mda-mb-435', 'all']	['mda-mb-435', 'NCI']	-mb-435', 'DrugCombDB']	['ovcar-8', 'all']	['ovcar-8', 'NCI']	vcar-8', 'DrugCombDB']	['pc-3', 'all']	['pc-3', 'NCI']	pc-3', 'DrugCombDB']	['sk-mel-5', 'all']	['sk-mel-5', 'NCI']	-mel-5', 'DrugCombDB']
Med	0.99	0.98	0.98	0.98	0.97	0.98	0.98	0.98	0.98	0.98	0.98	0.98	0.98	0.97	0.98	0.98	0.98	0.98
Mad	0.00	0.00	0.00	0.00	0.00	0.00	0.00	0.00	0.00	0.00	0.00	0.00	0.00	0.00	0.00	0.00	0.00	0.00
Mean	0.99	0.98	0.99	0.98	0.98	0.98	0.98	0.98	0.98	0.98	0.98	0.98	0.98	0.98	0.98	0.98	0.98	0.98
Std	0.00	0.00	0.00	0.00	0.00	0.00	0.00	0.00	0.00	0.00	0.00	0.00	0.00	0.00	0.00	0.00	0.00	0.00
Min	0.98	0.98	0.98	0.98	0.97	0.98	0.98	0.98	0.98	0.98	0.98	0.98	0.98	0.97	0.98	0.98	0.98	0.98
Max	0.99	0.99	0.99	0.99	0.98	0.99	0.99	0.99	0.99	0.99	0.99	0.99	0.98	0.98	0.98	0.99	0.99	0.99
Pct25	0.98	0.98	0.98	0.98	0.97	0.98	0.98	0.98	0.98	0.98	0.98	0.98	0.98	0.97	0.98	0.98	0.98	0.98
Pct75	0.99	0.98	0.99	0.98	0.98	0.98	0.98	0.98	0.98	0.98	0.98	0.98	0.98	0.98	0.98	0.98	0.98	0.98

Fig 21. Per-cell model PCC of NCI+DrugCombDB dataset for training round

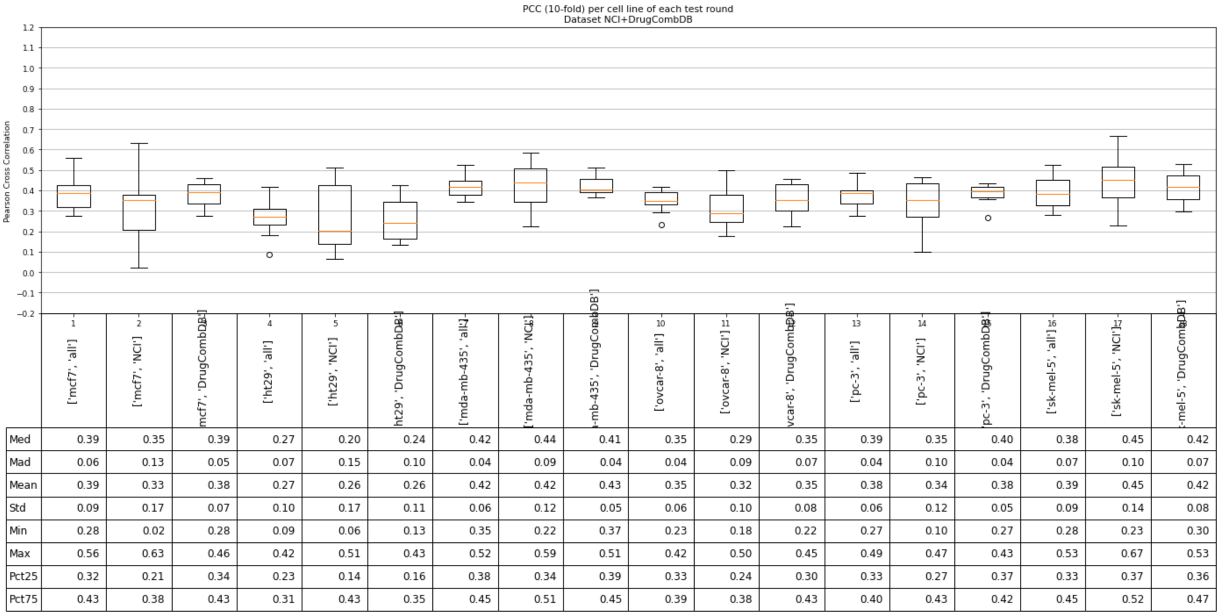

	['mcf7', 'all']	['mcf7', 'NCI']	mcf7', 'DrugCombDB']	['ht29', 'all']	['ht29', 'NCI']	ht29', 'DrugCombDB']	['mda-mb-435', 'all']	['mda-mb-435', 'NCI']	-mb-435', 'DrugCombDB']	['ovcar-8', 'all']	['ovcar-8', 'NCI']	vcar-8', 'DrugCombDB']	['pc-3', 'all']	['pc-3', 'NCI']	pc-3', 'DrugCombDB']	['sk-mel-5', 'all']	['sk-mel-5', 'NCI']	-mel-5', 'DrugCombDB']
Med	0.39	0.35	0.39	0.27	0.20	0.24	0.42	0.44	0.41	0.35	0.29	0.35	0.39	0.35	0.40	0.38	0.45	0.42
Mad	0.06	0.13	0.05	0.07	0.15	0.10	0.04	0.09	0.04	0.04	0.09	0.07	0.04	0.10	0.04	0.07	0.10	0.07
Mean	0.39	0.33	0.38	0.27	0.26	0.26	0.42	0.42	0.43	0.35	0.32	0.35	0.38	0.34	0.38	0.39	0.45	0.42
Std	0.09	0.17	0.07	0.10	0.17	0.11	0.06	0.12	0.05	0.06	0.10	0.08	0.06	0.12	0.05	0.09	0.14	0.08
Min	0.28	0.02	0.28	0.09	0.06	0.13	0.35	0.22	0.37	0.23	0.18	0.22	0.27	0.10	0.27	0.28	0.23	0.30
Max	0.56	0.63	0.46	0.42	0.51	0.43	0.52	0.59	0.51	0.42	0.50	0.45	0.49	0.47	0.43	0.53	0.67	0.53
Pct25	0.32	0.21	0.34	0.23	0.14	0.16	0.38	0.34	0.39	0.33	0.24	0.30	0.33	0.27	0.37	0.33	0.37	0.36
Pct75	0.43	0.38	0.43	0.31	0.43	0.35	0.45	0.51	0.45	0.39	0.38	0.43	0.40	0.43	0.42	0.45	0.52	0.47

Fig 22. Per-cell model PCC of NCI+DrugCombDB dataset for validating round

S2.3.2 training single global model with per-cell data combined

In this test, rather than training one model per cell line, we use the complete NCI dataset to train a single model. However, due to the size of the data being too large for both GPU memory and RAM, here we start with a subset of 6 randomly selected cell lines to charaterize the model training. The result shows that the behavior of such a global model is likely being dominated by several cell line data. The model performance of these dominating cell lines are over *PCC=0.65*. However, the model will ignore few cell lines which the model performance will drop below *PCC=0.5* (*cf. fig 17*).

For the training procedure, here we introduce a minor modification so cell line info can be coop with the training. Such step is necessary since without introducing cell line info, there can only be a single prediction output for the same drug pair. The modification is to introduce a numeric representation of cell line into the feature dimension (molecular fingerprint).

The *normal* feature dimension is **[molecular fingerprint]**, while the new feature dimension is **[cell ID]** + **[molecular fingerprint]** which **[cell ID]** is an arbitrary assigned incremental positive integer based similar to an ID. The cell id number would allow model to produce cell line based prediction.

In [4]:
```
import pandas as pd

df = pd.read_csv("R0008/cell_combined_data/cell_combined_data_agg_NCI.csv")
print(df)
```

```
       Unnamed: 0      ID        Drug1        Drug2   Cell line       ZIP  \
0               0   20051  Thioguanine    Pazopanib        MCF7 -3.222222
1               1   20052  Thioguanine   Raloxifene        MCF7 -9.000000
2               2   20053  Thioguanine  Abiraterone        MCF7 -0.666667
3               3   20055  Thioguanine   Vismodegib        MCF7  0.555556
4               4   20056  Thioguanine   Crizotinib        MCF7 -0.777778
...           ...     ...          ...          ...         ...       ...
16633       16633  146946  Oxaliplatin   Vismodegib  MDA-MB-435 -0.333333
16634       16634  146947  Oxaliplatin   Crizotinib  MDA-MB-435  4.555556
16635       16635  146948  Oxaliplatin    Axitinib  MDA-MB-435  3.777778
16636       16636  146949  Oxaliplatin   Vandetanib  MDA-MB-435  5.000000
16637       16637  146950  Oxaliplatin  Ruxolitinib  MDA-MB-435 -1.555556

          Bliss     Loewe       HSA
0     -3.222222 -3.222222 -3.222222
1     -9.000000 -9.000000 -9.000000
2     -0.666667 -0.666667 -0.666667
3      0.555556  0.555556  0.555556
4     -0.777778 -0.777778 -0.777778
...         ...       ...       ...
16633 -0.333333 -0.333333 -0.333333
16634  4.555556  4.555556  4.555556
16635  3.777778  3.777778  3.777778
16636  5.000000  5.000000  5.000000
16637 -1.555556 -1.555556 -1.555556

[16638 rows x 9 columns]
```

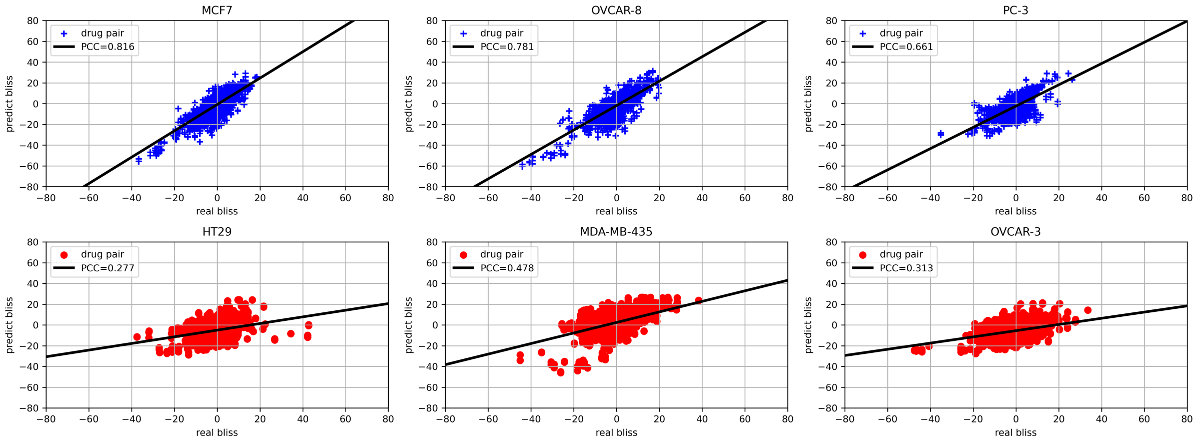

Fig 18. per-cell results of single model training with per-cell data combined. The model is demonstrating good performance for cell line MCF7, OVCAR-8 and PC-3 while lower performance for cell line MDA-MB-435, OVCAR-3 and HT29. The notable point in the figure is that the model performance is rather contrast for the pair of ovarian cancer cell lines namely OVCAR-3 vs OVCAR-8. In addition, MCF7 and MDA-MB-435 are both breast cancer cell lines while model performance is also very contrast.

In [ ]:
